# CURTAIN—A unique web-based tool for exploration and sharing of MS-based proteomics data

**DOI:** 10.1073/pnas.2312676121

**Published:** 2024-02-07

**Authors:** Toan K. Phung, Kerryn Berndsen, Rosamund Shastry, Tran L. C. H. B. Phan, Miratul M. K. Muqit, Dario R. Alessi, Raja S. Nirujogi

**Affiliations:** ^a^Medical Research Council Protein Phosphorylation and Ubiquitylation Unit, School of Life Sciences, University of Dundee, Dundee DD1 5EH, United Kingdom; ^b^Aligning Science Across Parkinson’s Collaborative Research Network, Chevy Chase, MD 20815

**Keywords:** proteomics, PTM, mass spectrometry, bioinformatics, sharing data

## Abstract

To facilitate analysis and sharing of mass spectrometry (MS)-based proteomics data, we created online tools called CURTAIN (https://curtain.proteo.info) and CURTAIN-PTM (https://curtainptm.proteo.info) with an accompanying series of video tutorials (https://www.youtube.com/@CURTAIN-me6hl). These are designed to enable non-MS experts to interactively peruse volcano plots and deconvolute primary experimental data so that replicates can be visualized in bar charts or violin plots and exported in publication-ready format. They also allow assessment of overall experimental quality by correlation matrix and profile plot analysis. After making a selection of protein "hits", the user can analyze known domain structure, AlphaFold predicted structure, reported interactors, relative expression as well as disease links. CURTAIN-PTM permits analysis of all identified PTM sites on protein(s) of interest with selected databases. CURTAIN-PTM also links with the Kinase Library to predict upstream kinases that may phosphorylate sites of interest. We provide examples of the utility of CURTAIN and CURTAIN-PTM in analyzing how targeted degradation of the PPM1H Rab phosphatase that counteracts the Parkinson’s LRRK2 kinase impacts cellular protein levels and phosphorylation sites. We also reanalyzed a ubiquitylation dataset, characterizing the PINK1-Parkin pathway activation in primary neurons, revealing data of interest not highlighted previously. CURTAIN and CURTAIN-PTM are free to use and open source, enabling researchers to share and maximize the impact of their proteomics data. We advocate that MS data published in volcano plot format be reported containing a shareable CURTAIN weblink, thereby allowing readers to better analyze and exploit the data.

## Significance

To facilitate the analysis of mass spectrometry-based proteomics data, we have generated free-to-use interactive tools termed CURTAIN and CURTAIN-PTM. These enable users to save analyzed proteomics data as an interactive and customizable volcano plot which can be shared with a weblink that can be included in publications. This permits other researchers to visualize and further analyze data contained within the volcano plots. CURTAIN and CURTAIN-PTM serve as a repository for mass spectrometry–based proteomics data as well as an easy-to-use interactive analysis and visualization tool, allowing researchers to maximize the impact of their data. To help users familiarize themselves with the tool, we have created a series of video tutorials available on a dedicated YouTube channel (https://www.youtube.com/@CURTAIN-me6hl).

Mass spectrometry (MS)-based proteomics is the go-to method to analyze proteomes and posttranslational modifications and is transforming all areas of biological investigation (1). Recent advances in sample preparation methodologies, separation and detection including nano-liquid chromatography and ultrasensitive MS instrumentation, enable the identification and quantification of >10,000 protein groups in a single-shot injection of peptides derived from cells or tissue extracts (2–4). MS-based proteomics has hugely impacted our understanding of signal transduction pathways by enabling accurate pinpointing and quantification of global patterns of posttranslational modifications including protein phosphorylation (5, 6), glycosylation (7), acetylation (8), and ubiquitylation (9). Recently developed, trapped ion-mobility-coupled time-of-flight MS platforms, make it possible to undertake ultrasensitive proteomics even at a single-cell level (10). Most MS data are now being acquired in a data-dependent (DDA) or data-independent acquisition (DIA) mode generating half a million MS and MS/MS events that can span more than 10 GB per file (4).

Differential expression proteomics data is commonly presented in a static volcano plot format (11). These are routinely generated by an MS expert following processing of the primary MS (raw) data from all replicates and experimental conditions, employing a sophisticated suite of analysis software including MaxQuant (12), MS-Fragger (13), Spectronaut (14), or DIA-NN (15). The output from these programmes are complex text files that are subsequently analyzed in statistical analysis programs such as Perseus (16), that does not require significant coding expertise. Other frequently used statistical packages such as MSstats (17) rely on the use of scripts and packages from Python and R.

The output from this analysis, that is routinely shared or submitted to journals for publication, is complex spreadsheets containing thousands of lines and dozens of columns together with non-interactive visualizations such as volcano plots. These volcano plots frequently contain a vast amount of useful information, and it is usually only possible for the presenter to highlight a small subset of the data that is of particular interest to them. Much data that could be of interest to other researchers is not highlighted nor straight-forward to visualize. It is challenging for the non-MS expert to subsequently retrieve and reanalyze the published data contained within a volcano plot image, and analyze the sets of proteins that they are most interested in. This requires access to the algorithm and statistical analysis output files and typically such analysis needs to be performed by a suitably trained expert. Analysis of MS data requires access to sophisticated software packages; many are neither free to use nor open-source, and need to be downloaded on a high performance computer. It is also demanding for the nonexpert to deconvolute the data presented on a volcano plot so that the primary data from each of the replicate experimental samples can be visualized and further analyzed.

Similar issues arise in analyzing volcano plots from posttranslational modification (PTM) analysis. It is frequently necessary to explore all the experimental PTM data available for a protein of interest, within each dataset. It is also important to visualize the location of each identified PTM on the protein(s) of interest and see how these modifications are impacted by experimental conditions. Moreover, it is necessary to be able to easily compare experimental PTM data with publicly available PTM databases such as PhosphoSitePlus (18), Protein Lysine Modification Database (PLMD) (19), CarbonylDB (20), GlyConnect (21), and Uniprot (https://www.uniprot.org/) (22). Finally, it is useful to analyze the amino acid sequence motifs encompassing each identified PTM.

To address the above issues, we have generated two open-source software, interactive tools termed CURTAIN (https://curtain.proteo.info) and CURTAIN-PTM (https://curtainptm.proteo.info). CURTAIN and CURTAIN-PTM necessitate no local computer server installation and operate through the internet on any computer equipped with a modern web browser. These tools are designed to provide users the option to save their analysis sessions with a weblink, enabling other researchers to visualize and further analyze these datasets interactively. These links can also be reported in publications allowing readers to easily explore and verify the quality of the reported data. We provide examples of the utility of CURTAIN and CURTAIN-PTM, by analyzing how targeted degradation of the Rab phosphatase, PPM1H, that counteracts the Parkinson’s LRRK2 kinase (23, 24), impacts on cellular protein levels using CURTAIN, and phosphorylation sites employing CURTAIN-PTM. To further demonstrate the utility of CURTAIN-PTM in analyzing ubiquitylation data, we have reanalyzed a previously reported dataset (25), characterizing how ubiquitylation of mitochondrial proteins is regulated by the Parkin E3 ligase. This reanalysis revealed interesting data not highlighted in the previous study. We discuss benefits for the research community of publishing proteomic data containing a shareable weblink, allowing readers to better explore data with open-source tools such as CURTAIN or CURTAIN-PTM.

## Results

### CURTAIN, A Web-based Tool for Visualization and Analysis of MS-based Proteomics Sata.

**Fig. 1.**
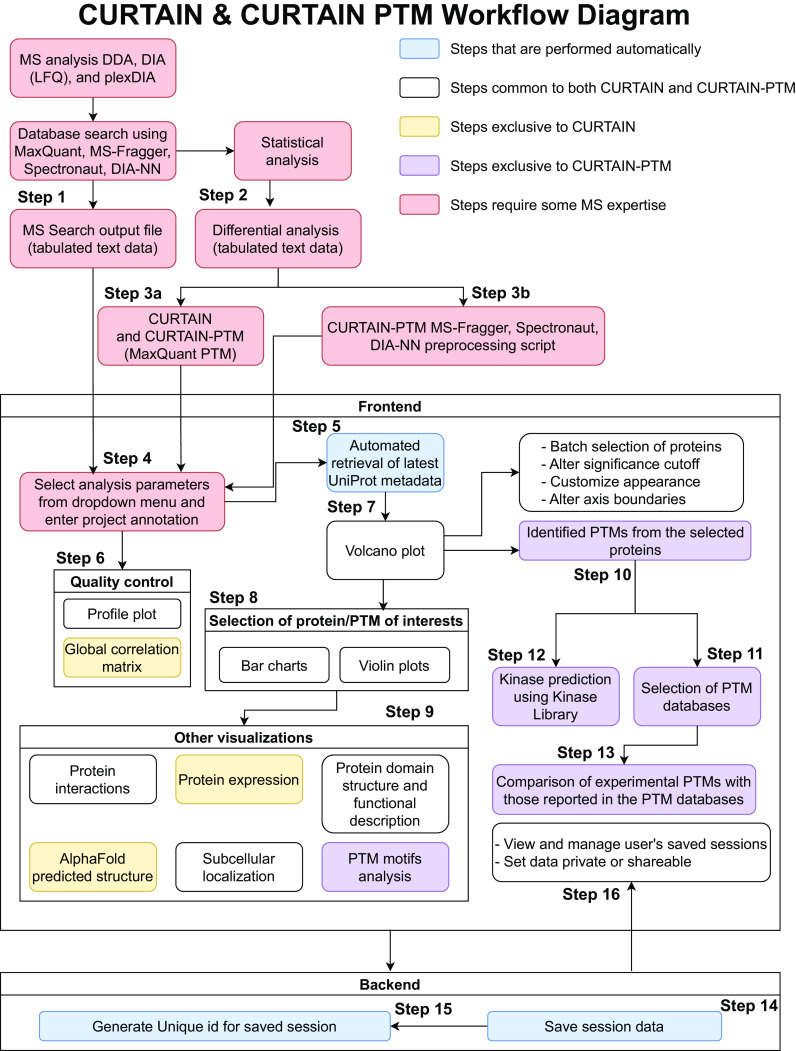
Workflow for importing, analyzing, and exploring processed MS-based total proteomics data with CURTAIN and CURTAIN-PTM. For further details, refer to the *Methods* and *Results* sections.

To facilitate analysis and sharing of complex MS data, we have created a web-based open-source tool called CURTAIN (https://CURTAIN.proteo.info/#/, RRID: SCR_024465), which is accompanied by a series of video tutorials (https://www.youtube.com/@CURTAIN-me6hl) demonstrating how to upload data and make the most of CURTAIN functionalities to customize and export plots, assess data quality, and explore the biology of experimental hits. The detailed front and backend framework of CURTAIN is described in the *Methods* section and summarized in Fig. 1. It allows interactive exploration of proteomic datasets which can be shared via a weblink. CURTAIN is designed to be used by non-MS-expert end users, once the analyzed MS data are uploaded by the MS expert who undertook the experiment.

CURTAIN requires two MS input files to be uploaded, which we recommend is undertaken by the MS expert who undertook the study. The first file to upload is the database search output file, in tabulated text format, of the primary MS data obtained from either MaxQuant (12), MS-Fragger (13), Spectronaut (14), or DIA-NN (15) in either data-dependent acquisition (DDA) (LFQ and TMT) as well as data-independent acquisition (DIA) (LFQ and plexDIA) modes (26) (step 1, Fig. 1). Second is a differential analysis file also in tabulated text format, that is obtained from statistical analysis programs such as Perseus, (16) MSstats (17) or an equivalent package (step 2, Fig. 1). In addition, the file columns to be analyzed and compared in the input file need to be selected and this is undertaken using a dropdown menu (step 4, Fig. 1, Dataset S1, and *SI Appendix*, Fig. S2*A*). At this stage, the metadata associated with the experimental data can be uploaded in the same format as used by the PRIDE database including project title, project description, sample processing protocol, data processing protocol, MS instrumentation details, and name and affiliations of researchers involved in the project (27) (step 4, Fig. 1 and *SI Appendix*, Fig. S2*B*). On opening a file, CURTAIN automatically retrieves the latest Uniprot metadata for each protein within the uploaded data (step 5, Fig. 1). At this stage, we recommend that the MS expert sends a weblink to end users to enable them to explore and analyze the data. All the subsequent steps can be readily performed by the nonspecialist end user.

**Fig. 2.**
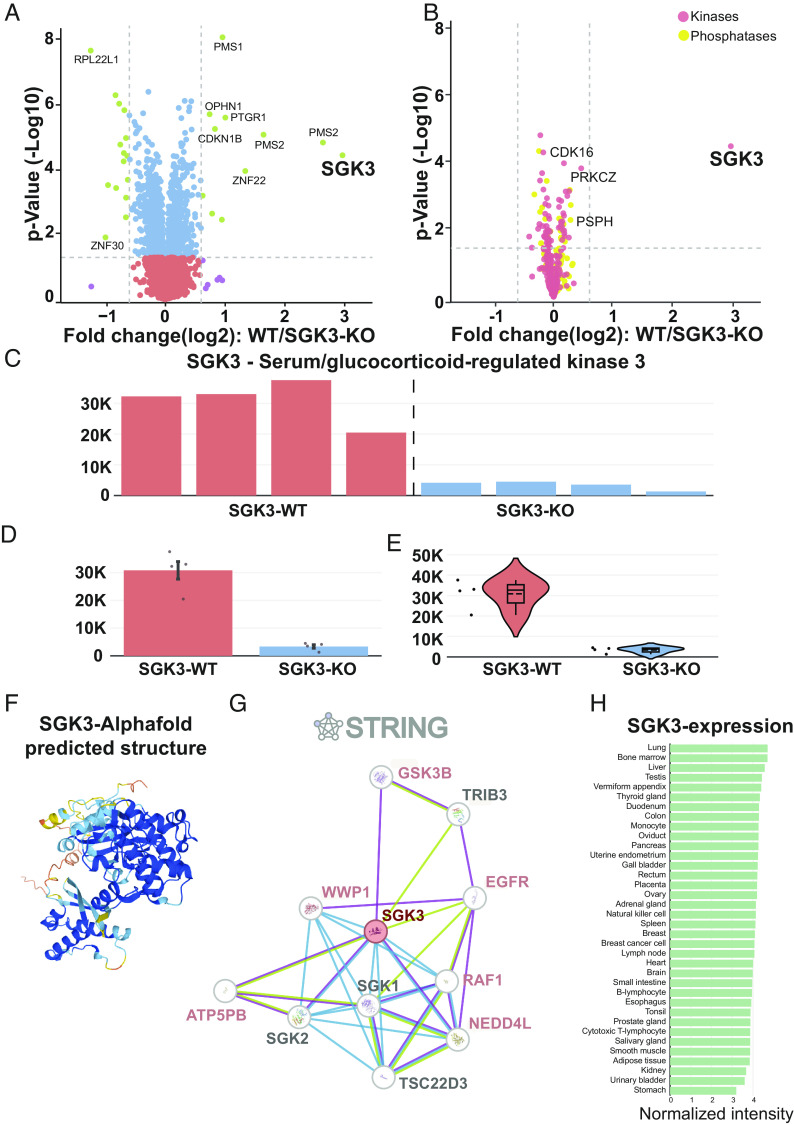
Functionalities of the CURTAIN tool. (*A*) Example of an interactive CURTAIN volcano plot employing previously reported proteomic data comparing wild-type (n = 4) and SGK3 (n = 4) knock-out HEK293 cells (PRIDE dataset identifier: PXD014561) (28). These data can be perused using the weblink h​​​​​​​​​​​ttp​s:/​/cu​rta​in.​pro​teo​.in​fo/​#/b​9e8​5c9​e-5​d26​-41​f2-​​847​4-1​8e3​985​fa804. The colored filled dots denote the ​sig​nif​icance and fold change cutoff values (red: ​non​sig​nif​icant and no ​dif​fer​ence; blue: ​sig​nif​icant and no ​dif​fer​ence; purple: ​non​sig​nif​icant but show ​inc​rea​se/​dec​rease in protein levels; and green: significant and show increase/decrease in protein levels). (*B*) Batch analysis of selected sets of proteins with irrelevant proteins faded https://curtain.proteo.info/#/aab22416-663a-40c3-a2e6-1839ff0d4dbd. (*C*–*H*) Deconvolution of primary experimental data for the SGK3 hit showing as individual bar graphs (*C*) or combined average bar graph (±SEM or SD) (*D*) or violin plot (*E*), https://curtain.proteo.info/#/4e792c03-099b-4b1b-bd4e-18afe4204f2c. (*F*) Predicted AlphaFold structure of SGK3. (*G*) Reported STRING SGK3 interactors with gene names are color coded to depict the presence/absence within the experiment and increase/decrease in fold-change levels (dark red: detected/increase in expression; blue: detected/decrease in expression; light red: detected/NO-difference; and gray: not detected in the dataset). (*H*) Protein expression data of SGK3 derived from ProteomicsdB.

CURTAIN enables end users to assess the quality of proteomic data by generating profile plots (*SI Appendix*, Fig. S3*A*) and a global correlation matrix (*SI Appendix*, Fig. S3*B*) of uploaded data (step 6, Fig. 1). On the profile plots, the relative levels of specific proteins of interest can be displayed across all samples (*SI Appendix*, Fig. S3*A*). CURTAIN displays the differential analysis data from each set of experiments as an interactive volcano plot displaying the fold-difference (*x* axis, typically in Log2) and *P*-value significance (*y* axis, typically in −log10) (step 7, Figs. 1 and 2*A*). The end user can then customize the volcano plot display color including color blind friendly options, labeling, annotation (including font size and color selection) and altering axis boundaries (step 7, Figs. 1 and 2*B*). It also allows modification of fold change and significance cutoff to be represented by vertical and horizontal dotted lines, respectively (step 7, Figs. 1 and 2 *A* and *B*). In the default settings, hits that pass the cutoff values are represented in a different color, but this can be modified as required.

For more in depth analysis, the end user selects data of interest, for example, a set of proteins which are changing between experimental conditions (step 8, Figs. 1 and 2*B*). For each selected protein, CURTAIN allows visualization of the primary data for all replicates for each condition directly as a bar chart either for separate (step 8, Figs. 1 and 2*C*) or combined bars ± SEM or SD (Fig. 2*D*). It also allows data to be presented as a violin plot format ± SEM or SD (step 8, Figs. 1 and 2*E*).

Another feature of CURTAIN is that it provides the option for users to learn more about the identified proteins (step 9, Fig. 1). For all selected hits, CURTAIN allows visualization of the Uniprot functional domain structure which also provides a concise functional description of what is known about the selected protein as well as predicted/known subcellular localization (22), AlphaFold predicted structure (29) (Fig. 2*F*), and known or predicted interactors derived from STRING (30) (Fig. 2*G*). Another feature of the interactome database is that it reports interactors of selected hits that are identified within the uploaded data, highlighting proteins whose levels change between experimental conditions (red for higher, blue for lower, pink present in dataset but levels unchanged and black not detected in dataset) (Fig. 2*G*). It is possible to search the Interactome Atlas (31) to find interactors of the selected protein, and to view its expression profile across different cell and tissue types using ProteomicsDB (32) (Fig. 2*H*). CURTAIN will also link selected hits to the disease, pharmaceutical and mutagenesis data available within the Uniprot database.

CURTAIN also allows for the facile batch selection of a group of proteins of interest to the end users. We have separated these into 4 categories, namely diseases (e.g., Parkinson’s, Alzheimer’s), enzymes (e.g., kinases, phosphatases), organelles (e.g., Golgi, lysosomes), and pathways (e.g., LRRK2, PINK1), that are listed in Dataset S2, and these can readily be updated and added to. CURTAIN also allows users to select groups of proteins within their dataset or more generally that are of particular interest and to save these as a set to facilitate further analysis of these proteins. To further facilitate viewing of selected proteins of interest, CURTAIN permits “hiding” of all other nonselected proteins in the Volcano plot (Fig. 2*B*).

Each analysis session on CURTAIN can also be saved and shared with a unique weblink (step 14 and step 15, Fig. 1). The person(s) receiving this link can further analyze and download the data. All graphical outputs from CURTAIN can be exported in SVG format and further modified in programs such as Adobe Illustrator. Weblinks to the CURTAIN data including volcano and violin plots can be included in the figure legend of publications allowing readers to easily access, view, and analyze the data further. The source code for CURTAIN can be downloaded in Github (*Methods*), hosted on a local server, and modified for additional functionalities. To help organize and keep track of different projects, CURTAIN allows users to login using ORCID, and all the different user sessions across projects are saved within the user account. Saved session data can be maintained private or shared with a weblink (Fig. 1). CURTAIN can also be used without account creation and sessions saved and retrieved using a unique weblink.

### CURTAIN-PTM for Visualization and Analysis of Posttranslational Modification Proteomics Data.

**Fig. 3.**
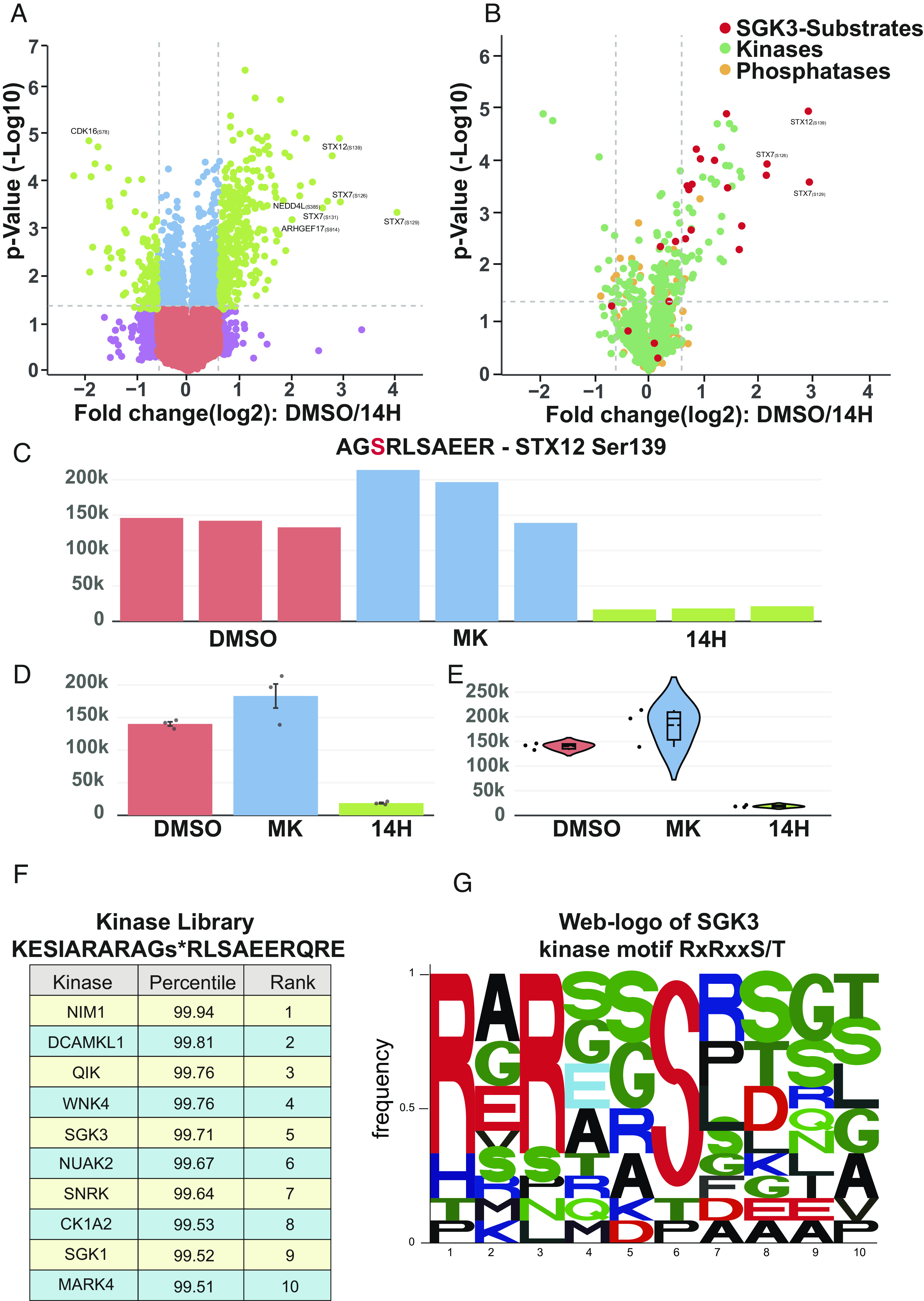
Functionalities of the CURTAIN-PTM tool. (*A*) Example of an interactive CURTAIN-PTM volcano plot using previously reported phospho-proteomic data of wild-type HEK293 cells that had been serum starved overnight and then treated with either no inhibitor (DMSO control n = 3) or a selective pan Akt isoform inhibitor (1 M MK-2206, MK abbreviation, 1 h, n = 3) or a pan SGK isoform inhibitor (3 µM 14H, 1 h, n = 3) prior to stimulation with IGF-1 (50 ng/mL, 15 min) in the continued presence of the inhibitor (PRIDE dataset identifier: PXD014561) and data analyzed using MaxQuant (28) https://curtainptm.proteo.info/#/d79cff8f-cd8f-4f4a-b082-7f7807f8a3d4. (*B*) Batch analysis of selected sets of phospho-sites with irrelevant phospho-sites faded https://curtainptm.proteo.info/#/a1566c36-91f3-45e2-b955-881194681ba5. (*C*–*G*) The SGK3 substrate, STX12 (Ser139), was selected for further analysis. The relative levels of STX12 (Ser139) in each sample were viewed in individual bar graphs (*C*) or combined average bar graph (±SEM or SD) (*D*) or violin plot (*E*) https://curtainptm.proteo.info/#/d79cff8f-cd8f-4f4a-b082-7f7807f8a3d4. (*F* and *G*) The sequence encompassing the Ser139 site on STX12 submitted to the Kinase Library database to predict kinases that may phosphorylate this residue (33) (*F*) or the weblogo tool (34) (*G*).

To analyze MS-based posttranslational modification (PTMs) data, we have also developed CURTAIN-PTM (https://curtainptm.proteo.info/#/, RRID: SCR_024465), which is accompanied by two instructional video tutorials (https://www.youtube.com/@CURTAIN-me6hl). CURTAIN-PTM deploys the same backend as CURTAIN. The search output and differential files containing PTM information are uploaded (Fig. 1, steps 1 to 3). Currently, CURTAIN-PTM is optimized to use output from MaxQuant PTM, both DDA (LFQ and TMT) and DIA (LFQ) mode. It does require preprocessing of output PTM data files from MS-Fragger, Spectronaut, and DIA-NN by employing CURTAIN utility python package (https://doi.org/10.5281/zenodo.10079224); this step can be best undertaken by an MS expert (Fig. 1, step 3b). For our PTM site analysis, we normally upload class-I, high confidence sites (>0.75 probability), but it is possible to upload files containing lower probability sites if required. CURTAIN-PTM displays the differential PTM analysis data from each set of experiments as an interactive volcano plot that can be further analyzed, modified, exported, and saved as described for CURTAIN (Fig. 3*A*). The end user selects a set of modified peptide(s) (e.g., phosphorylated or ubiquitylated) of interest, for example, those whose levels are modulated between experimental conditions (Fig. 3*B*).

CURTAIN-PTM also allows for the deconvolution and visualization of the primary data for all replicates of each condition as a bar chart with error bars (±SEM or SD) (Fig. 3 *C* and *D*) or violin plot format (Fig. 3*E*). For phosphorylation site analysis, CURTAIN-PTM links to Kinase Library (https://kinase-library.phosphosite.org/site) (33), thus providing predictions for kinases that may phosphorylate this selected site, based on sequence analysis and knowledge of kinase substrate specificities (Fig. 3*F*). The user can also select a group of peptides for sequence motif analysis using the weblogo tool (Fig. 3*G*).

For each selected peptide, CURTAIN-PTM provides the gene name along with the PTM residue number(s) derived from the search algorithm (Fig. 3*A*). CURTAIN-PTM also allows the user to link back to the protein from which it is derived, allowing for all experimentally identified PTMs found within that protein to be visualized on a linear protein sequence and compared against selected publicly available databases (Fig. 1 steps 10, 11, and 13, *SI Appendix*, Fig. S4*A*). The Uniprot database (22) is the default option, but other databases including PhosphoSitePlus (18) can also be selected (Dataset S3).

It should be noted that the residue numbering of PTMs obtained from MaxQuant and other analysis packages depends on the splice variant reported by the search algorithm, which may differ from the canonical sequence used by other databases or commonly discussed in the literature. CURTAIN-PTM lists numbering based on that used by the original search algorithm and annotates this as “experimental data” (*SI Appendix*, Fig. S4*A*). CURTAIN-PTM then performs a multiple sequence alignment between the identified PTM site and the canonical isoform listed on the selected database (*SI Appendix*, Fig. S4*A*). If there is a difference in sequence between the Uniprot and experimental sequence due to splice variant numbering, CURTAIN-PTM displays the sequence alignment and provides the selected database PTM residue number in addition to the experimental residue position (*SI Appendix*, Fig. S4*B*). When other PTM databases are selected for comparison with the experimental data, the PTM residue number derived from the selected database(s) will be displayed. Some databases such as PhosphoSitePlus enable user-selected splice variants to be analyzed. CURTAIN-PTM color codes residue positions: purple indicates that the experimental data overlap with the PTM identified in the selected database(s), red is that the experimental PTM is not observed in the selected database(s), blue depicts data that are present in the selected databases, but not in the experimental data, and a peptide selected for further analysis is displayed in green (*SI Appendix*, Fig. S4). Sites that change significantly between experimental conditions are highlighted with an asterisk (*SI Appendix*, Fig. S4).

CURTAIN-PTM also permits custom PTM database usage in the form of a tabulated text file. To demonstrate this, we have used a published dataset containing protein citrullination (35) and generated a custom citrullination PTM database which can be viewed within the CURTAIN-PTM tool for any selected hit of interest (*SI Appendix*, Fig. S5). The custom database can be named by the user and viewed in the PTM viewer. It should be noted that there are limitations to the file size of the database that can be imported depending on the user’s browser and operating system.

### Example of Use of CURTAIN and CURTAIN-PTM to Analyze Proteomic and Phosphoproteomic Analysis.

**Fig. 4.**
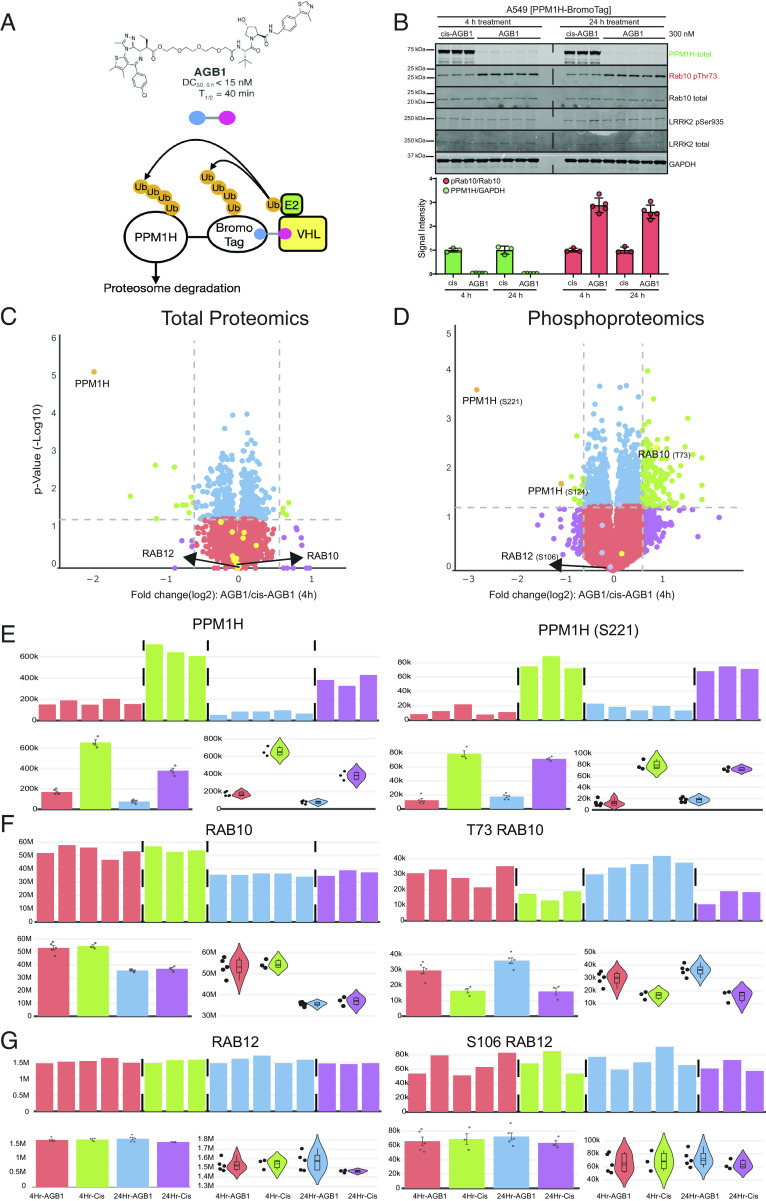
Use of CURTAIN and CURTAIN-PTM to analyze the impact of cellular depletion of the PPM1H protein phosphatase. (*A*) Schematic depicting the mechanism by which the AGB1 compound induces the targeted degradation of the CRISPR knock-in PPM1H-BromoTag. (*B*) Knock-in A549 PPM1H-BromoTag cells were treated with 300 nM cis-AGB1 (inactive control compound) or AGB1 for 4-h or 24-h prior to cell lysis. Then, 20 μg whole cell lysate was subjected to quantitative immunoblot analysis with the indicated antibodies. Membranes were developed by the LICOR Odyssey CLx Western Blot imaging system. Quantitation of the ratio of phospho-Thr73/total Rab10 and PPM1H/GAPDH was obtained using Image Studio software. Each lane represents lysate obtained from a different dish of cells. Individual data points of the replicates within the figure are shown, with the error bars representing the SD of the mean between the replicates. (*C*) The lysates produced from the experiments described in Fig. 4*B* in which cells were treated for 4-h with cis-AGB1 or AGB1 were subjected to total and phospho-proteomic analysis, with data being analyzed with MS-Fragger and MaxQuant. CURTAIN-generated volcano plots are presented for the total proteomic (*C*, https://curtain.proteo.info/#/f4b009f3-ac3c-470a-a68b-55fcadf68d0f) and Phospho-proteomic (*D*, https://curtainptm.proteo.info/#/32837924-c5bb-41a2-9d30-b5839c012243) and proteins of interest highlighted in black. The same color coding is used as described in Fig. 3*A*. (*E*–*G*) The primary total and phosphosite intensities of indicated hits were shown as bar graphs and violin plots.

To demonstrate the utility of the CURTAIN and CURTAIN-PTM tools, we employed an inducible protein degradation strategy termed the BromoTag (36), to rapidly deplete the endogenous levels of a protein phosphatase termed PPM1H, that was previously shown to dephosphorylate the Rab10 substrate that is phosphorylated by the LRRK2 protein kinase implicated in Parkinson’s disease (23). The degron BromoTag was linked to the C terminus of PPM1H using CRISPR/Cas9 gene-editing technology as the NH terminus of this enzyme is involved in membrane binding (24) (Fig. 4*A*). Homozygous knock-in cell lines were selected and verified by immunoblot and DNA-sequencing analysis (*SI Appendix*, Fig. S6 *A*–*D*). Homozygous PPM1H-BromoTag knock-in cells were treated for 4-h or 24-h with the selective degrader compound termed AGB1, or as a control, the structurally similar inactive cis-AGB1 analogue (36). Depleting the PPM1H phosphatase would be expected to result in an increased phosphorylation of its physiological Rab10 substrate. Immunoblot analysis confirmed that PPM1H-BromoTag levels were reduced by >95% with the AGB1 compared to the cis-AGB1-treated cells within 4-h (Fig. 4*B*). As expected, this was accompanied by an increase in pRab10 levels as judged by immunoblotting with a phospho-specific antibody (Fig. 4*B*). As expected, the total levels of Rab10, LRRK2, and LRRK2 biomarker phosphosite pSer935 were unaffected by AGB1 treatment (Fig. 4*B*).

We next undertook a 16-plex tandem mass tags (TMT)-based quantitative proteomic and phosphoproteomic analysis as described in *Methods*, of PPM1H-BromoTag A549 cells treated with AGB1 and cis-AGB1 for 4- and 24-h. The MS proteomic data were searched using the MS-Fragger algorithm (13) which identified 8,609 unique proteins (Dataset S4). The phosphoproteomic data were analyzed using MaxQuant (12) which identified and quantified 31,808 phosphosites and 25,203 class-I phosphosites (Dataset S4). Statistically significant differentially regulated proteins and phosphosites were determined using Perseus (16). The result files from these analyses were uploaded to CURTAIN and CURTAIN-PTM using the parameters described in Dataset S1. CURTAIN-generated profile plots revealed the same distribution of proteins in each sample, indicating high-quality data (*SI Appendix*, Fig. S7*A*). Levels of Rab10 were similar in all samples, whereas as expected, the levels of PPM1H were reduced in AGB1 compared to cis-AGB1-treated cells (*SI Appendix*, Fig. S7*A*). A CURTAIN-generated global correlation matrix revealed ~0.98 correlation between each replicate in the different experimental conditions lysed at the same time points (*SI Appendix*, Fig. S7*B*). Cells lysed at different time points displayed a reduced 0.96 correlation suggesting slight batch effects which are to be expected (*SI Appendix*, Fig. S7*B*). CURTAIN generated volcano plots for both the AGB1 4-h (Fig. 4*C*, CURTAIN, Fig. 4*D*, CURTAIN-PTM) and 24-h (*SI Appendix*, Fig. S8*A*-CURTAIN, *SI Appendix*, Fig. S8*B*-CURTAIN-PTM) time points. As expected, PPM1H was the protein most significantly reduced by AGB1, at both the 4-h and 24-h time points (Fig. 4*C* and *SI Appendix*, Fig. S8*A*). Analysis of the primary PPM1H protein data confirms a marked reduction of PPM1H levels in all replicates for AGB1-treated samples (Fig. 4*E*). A number of other proteins were moderately impacted by AGB1, and CURTAIN-generated violin plots for these are shown in *SI Appendix*, Fig. S9. Batch selection of known LRRK2 pathway proteins suggests that other than PPM1H, other known pathway components were unchanged (yellow circles, Fig. 4*C*). Based on the cutoff values used, phosphosite analysis revealed that the levels of S221 and S124 phosphosites were enhanced at the 4-h (Fig. 4*D*) and 24-h (*SI Appendix*, Fig. S8*B*) time points, respectively. As expected from the immunoblotting data, the phosphopeptide encompassing the LRRK2 Rab10 phosphorylation site (pThr73) is enhanced ~twofold at both the 4-h and 24-h time points of AGB1 treatment (Fig. 4*D*). LRRK2 also phosphorylates Rab12 at Ser106, but previous data suggest that PPM1H does not regulate dephosphorylation of Rab12 in vivo (23) as these proteins localize at different sites within the cell (24). Consistent with this, levels of the phosphopeptide encompassing Rab12 phosphorylated at Ser106 were not altered in PPM1H-depleted cells (Fig. 4*G*). Violin plots of other selected phosphorylation sites that were most enhanced in the PPM1H-depleted cells at both 4-h and 24-h time points is presented in *SI Appendix*, Fig. S10. These results illustrate how CURTAIN-PTM can mine phospho-proteomics data. Further work is required to establish whether the additional identified phosphosites represent direct PPM1H substrates.

### Example of Use of CURTAIN-PTM to Analyze Ubiquitylation Sites.

**Fig. 5.**
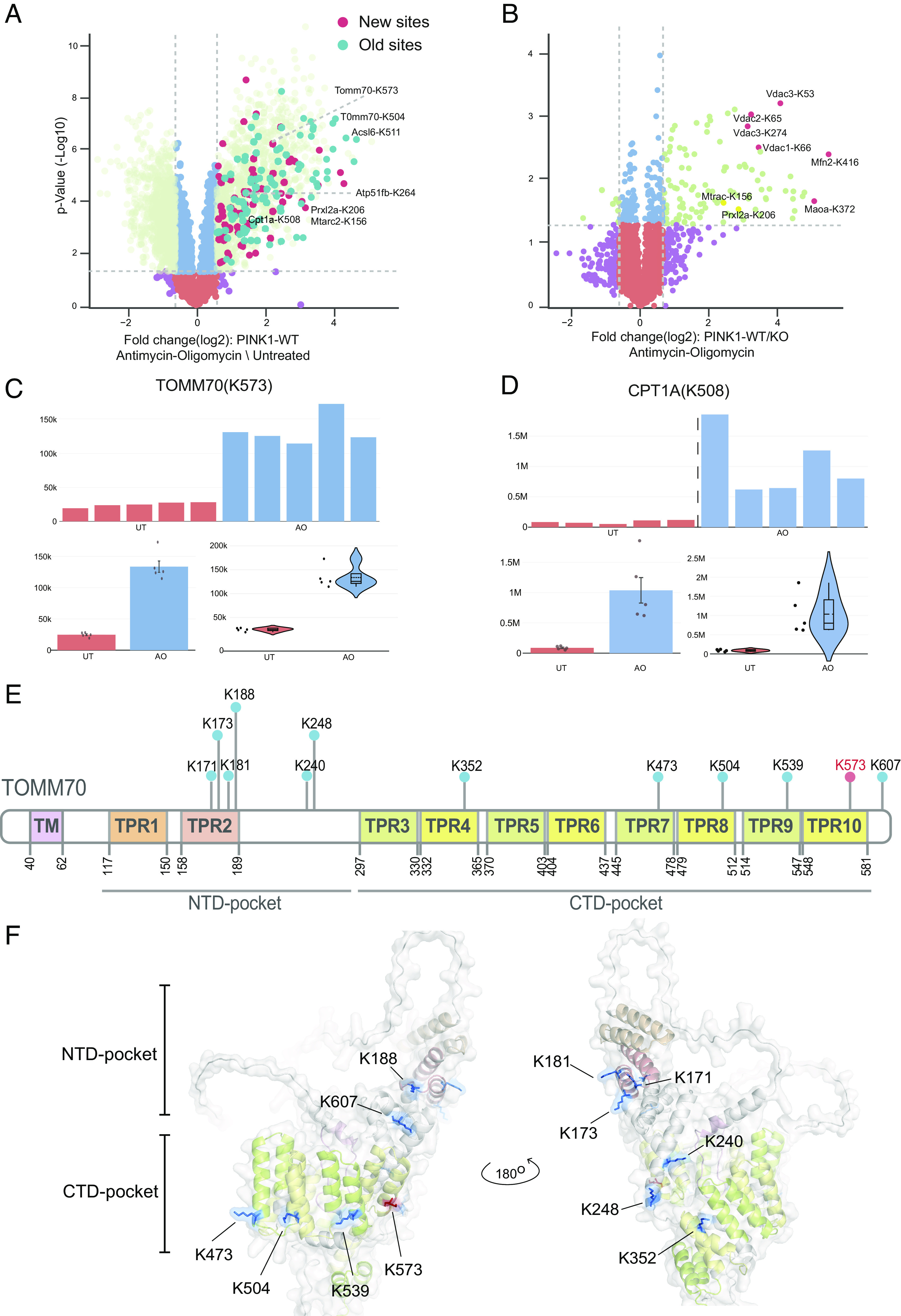
Visualization of Parkin-mediated ubiquitylation in neurons using CURTAIN-PTM. The MS raw data from a previously published study (MassIVE dataset identifier MSV000087639) (25) were reanalyzed using the MS-Fragger search algorithm. (*A*) Wild-type C57BL/6J primary cortical neurons were depolarized ± Antimycin A (10 μM)/Oligomycin (1 μM) (AO) stimulation for 5 h or (*B*) wild-type and PINK1 knock-out neurons were depolarized ± AO for 5 h. Membrane-enriched lysates were subjected to diGLY capture proteomics as described previously (25). CURTAIN-PTM generated volcano plots https://curtainptm.proteo.info/#/85970b1d-8052-4d6f-bf67-654396534d76 (*A*) https://curtainptm.proteo.info/#/cffbae16-abb0-418d-8244-801e7bd46bc0 (*B*). The color coding is described in Fig. 3*A*, and new highlighted hits are in dark red and analyzed further in *C* and *D* (TOMM70-K573, CPT1A-K508) and *SI Appendix*, Fig. S11 *A* and *B*, while previously highlighted hits are shown in dark blue and analyzed in *SI Appendix*, Fig. S11 *C–E*. (*E*) Schematic representation of the domain arrangement of TOMM70 (M. musculus), highlighting the transmembrane region (TM) and TetraTricopeptide repeat (TPR) domains as annotated in UniProt. ubiquitylated lysines (K) are indicated. (*F*) Structural representation of TOMM70 (M. musculus) highlighting the localization of lysine residues (previously identified ubiquitin sites in blue and newly identified sites in red). The structure was acquired from AlphaFold and analyzed in PyMOL (version 2.5.2). The color scheme employed corresponds to the domains depicted in the schematic representation of 9.

To demonstrate the utility of CURTAIN-PTM in the analysis of ubiquitylation sites, we reanalyzed a previously published ubiquitylome dataset in which primary C57BL/6J mouse cortical neurons, were treated ± mitochondrial agents [10 μM Antimycin A/1 μM Oligomycin (AO) for 5 h] to stimulate ubiquitylation of mitochondrial proteins via the PINK1 kinase-Parkin E3 ligase signaling pathway (25). Tryptic peptides derived from mitochondrial enriched extracts were subjected to a diGLY immunoprecipitation step that enriches for ubiquitylated peptides which were subsequently quantified using a TMT-based approach (25) (Fig. 5*A*). Similar reanalysis was performed for a parallel dataset of wild-type (WT) and PINK1 knockout (KO) neurons (Fig. 5*B*) (25).

The ubiquitylation data were reanalyzed using the MS-Fragger search algorithm which identified and quantified ~6,000 ubiquitylation sites. Statistically significant differentially regulated ubiquitylation sites were determined using Perseus. The resultant files from these analyses were uploaded to CURTAIN-PTM using the parameters described in Dataset S5, and we generated a CURTAIN volcano plot for AO 5-h treatment versus untreated (Fig. 5*A*). This enabled visualization of previously reported AO-induced ubiquitylated sites (light green circles, Fig. 5*A*) including Parkin-dependent mitochondrial substrates (dark blue circles, Fig. 5*A*). Using CURTAIN-PTM, we also generated a volcano plot for AO 5-h-treated PINK1 WT versus PINK KO neurons depicting previously reported PINK1-dependent ubiquitylated sites including VDAC1 (K66) and Mfn2 (K416) (Fig. 5*B*). CURTAIN-PTM allows facile identification of all identified ubiquitylation sites within the uploaded dataset. Performing this analysis, we observed several ubiquitylation sites on proteins that were not previously reported including Atp51fb (K264), Mtarc2 (K156), and Prxl2a (K206) (red circles, Fig. 5*A*) and of which the latter two sites are PINK1-dependent (Fig. 5*B*). The additional sites were likely identified by our reanalysis of the original raw data using the MS-Fragger search algorithm that was not used in the original study. CURTAIN-PTM simplifies the detection and analysis of these additional sites. In future work, it would be interesting to assess whether Mtarc2 and Prxl2a are direct Parkin substrates. Furthermore, reanalysis elaborated new sites on previously reported Parkin substrates including TOMM70 (K573) (Fig. 5*C*) and CPT1A (K508) (Fig. 5*D*). In addition, other new ubiquitylated sites are shown in *SI Appendix*, Fig. S11 *A* and *B*, and previously described sites in *SI Appendix*, Fig. S11 *C–E*. CURTAIN-generated bar charts for CPT1A (K508) and TOMM70 (K573) ubiquitylation site levels confirmed an increase across all replicates of AO-treated neuron samples, and this is also confirmed by CURTAIN-generated violin plots for these sites. TOMM70 encodes a key subunit of the translocase of the outer membrane (TOM) and consists of multiple repeating units known as TetraTricopeptide repeat (TPR) domains. The N-terminal TPRs form a loosely structured region called the NTD-pocket, which primarily interacts with molecular chaperones, e.g., heat shock proteins (Fig. 5*E*). The C-terminal TPRs form the CTD-pocket, which specifically binds to mitochondrial preproteins for import into the mitochondria (Fig. 5*E*). Analysis of an AlphaFold structural model of mouse TOMM70 reveals that the previously reported Parkin-mediated ubiquitylation sites span multiple TPR domains of the NTD [(K171, K172, K181, K188, K240, and K248) and CTD pockets (K352, K473, K504, K539, K573, K607). Interestingly, the newly identified TOMM70 site (K573) lies within a common surface in the CTD-pocket forming a distinct ring-like pattern with the previously reported sites (K473, K504, and K539) (Fig. 5*F*).

## Discussion

We generated two unique tools CURTAIN and CURTAIN-PTM that can be used by a non-MS expert to explore complex MS-based quantitative proteomic and PTM data. These are free to use, open source, and web-based software requiring no installation. Both enable any end user to freely share and explore MS-based proteomic data with a unique weblink. To the best of our knowledge, there are no such free-to-use open-source software packages similar to CURTAIN and CURTAIN-PTM. The programs that are closest to CURTAIN are SimpliFi^™^ (https://protifi.com/pages/simplifi) (37), Mass Dynamics (https://massdynamics.com) (38), ProteoSign (http://bioinformatics.med.uoc.gr/ProteoSign/) (39), and POMAShiny (40). We have provided a spreadsheet that summarizes the features of these other tools compared to CURTAIN and CURTAIN-PTM (Dataset S7). Several of these tools are commercial or can only be used on a personal computer. These packages lack several of the features built into CURTAIN most importantly allowing data to be freely shared and analyzed by any end user. Moreover, none of the other tools we are aware of allow for PTM data visualization, analysis, and comparison with selected PTM databases. These tools do not allow users to generate curated batch searches of proteins of interest such as kinases, phosphatases, ubiquitin components, and groups of proteins linked to disease. The other tools mentioned do not also link to the predicted AlphaFold structure or Kinase Library that CURTAIN and CURTAIN-PTM links to. It should further be noted that CURTAIN and CURTAIN-PTM do not store the primary MS raw data and thus should be considered as a companion but not replacement to the PRIDE database (27). For immunological proteomic MS data, the ImmPRes (Immunological Proteome Resource, http://immpres.co.uk/) is widely used (41). This operates as a curated database with interactive visualization of deposited data but does not allow users to upload their own data to this and share links to enable others to explore, deconvolute, and analyze your proteomic data with a weblink. The ImmPRes database does not currently allow the analysis of PTM data.

CURTAIN-PTM is currently directly compatible with PTM data derived from MaxQuant search that maps the modified sites on peptides along with the site probability information and quantification. The search output for PTM data from other search algorithms such as MS-Fragger, Spectronaut, and DIA-NN is not directly fully compatible with CURTAIN-PTM as this displays the data differently from MaxQuant. For example, the output from Spectronaut displays the modified peptide without information on the residue number of the protein that it is derived from. Unlike MaxQuant, Spectronaut does not output separate PTM files for each modification and only provides a combined file that lists all of the modifications. DIA-NN provides the PTM site probability files and quantification data as different files which would need to be merged and processed prior to uploading into PTM. The output from MS-Fragger search [DDA-LFQ, TMT, and DIA, Thermo Raw files only (42)] does not provide the modification residue number within the peptide. To circumvent this, we have developed a python package (https://github.com/noatgnu/curtain-utils) to allow PTM data to be processed from MS-Fragger, Spectronaut, and DIA-NN in a format that is compatible with CURTAIN-PTM (Fig. 1, step 3B). In the future, we aim to automate these steps within CURTAIN-PTM.

MS-based proteomic and PTM data are normally generated and shared between collaborators and presented in journals in a static format either in Figures and/or Tables linked to the PRIDE database that hosts the raw MS data (27). These formats, especially for non-MS expert users, can be challenging to download and explore. This can limit the potential impact of MS datasets as crucial data that could be discovered and exploited by other researchers, remain buried within these noninteractive datasets. We think that CURTAIN and CURTAIN-PTM will significantly facilitate the exploration of published MS Proteomics and PTM data by nonexperts. This will also enable the quality and robustness of the MS data to be more readily assessed which is important if future research is to be based on this. We advocate that a sharable CURTAIN and/or CURTAIN-PTM weblink or equivalent package be reported in all figure legends of figures or tables displaying MS data, and we have done this for a recent study (43). CURTAIN and CURTAIN-PTM are also extremely useful in sharing MS data between lab members and collaborators. Altogether, these tools align with the FAIR principles to share experimental data (Findable, Accessible, Interoperable, and Reusable). Here, we have developed two tools that facilitate open science, open source, and ultimately replication of scientific advances (44).

Last, we have specifically designed CURTAIN and CURTAIN-PTM for proteomic and phosphoproteomic data but envisage adapting CURTAIN to analyzing other kinds of OMICs data including metabolomic, lipididomic, or RNA sequencing data, therefore expanding the potential impact of these analytical tools to different research fields and human diseases.

In conclusion, CURTAIN and CURTAIN-PTM are free-to-use open-source useful tools, enabling, sharing, and exploration of complex MS-based proteomics data. These are designed to democratize the exploitation of proteomic data by non-MS experts. We recommend that published studies involving proteomic experiments contain a shareable weblink, allowing reviewers and readers to explore the data interactively. This will help maximize the benefit and impact of published proteomics data.

## Materials and Methods

Detailed descriptions of materials and methods are available in *SI Appendix*.

### Methods.

CURTAIN was created using the Angular Web framework (https://angular.io/) and Python. The complete details and access to the code availability are in *SI Appendix*.

### Technical Implementations.

#### CURTAIN Frontend.

The workflow of CURTAIN is described in Fig. 1. The input selection parameters for CURTAIN are selected through an inbuilt dropdown menu (step 4, Fig. 1) and summarized in Dataset S1. Experimental metadata can be added in the same format as used by the PRIDE database (27) (step 4, Fig. 1). Retrieval of up-to-date Uniprot metadata (step 5, Fig. 1) uses the Uniprot web API (22). Visualization of the quality controls: profile plot, global correlation matrix (step 6, Fig. 1), volcano plot (step 7, Fig. 1), bar graphs/violin plots (step 8, Fig. 1), protein domain structure, and protein expression profile (step 9, Fig. 1) employs Plotly (https://plot.ly) (45). In step 9, Fig. 1, domain structures, disease links, mutagenesis information, and pharmaceutical use are determined using Uniprot (https://www.uniprot.org/) (22), predicted structure using AlphaFold (https://alphafold.ebi.ac.uk/) (29, 46), protein interactions deploying the STRING interaction database (https://string-db.org/) (30) as well as the Interactome Atlas (http://www.interactome-atlas.org/) (31) and tissue expression uses ProteomicsDB (https://www.proteomicsdb.org/) (32).

#### CURTAIN-PTM Frontend.

CURTAIN-PTM is designed using the same framework described above. The workflow of CURTAIN-PTM is described in Fig. 1. Steps 1 to 8 in CURTAIN-PTM are the same as CURTAIN, except in step 3b, preprocessing of PTM data derived from MS-Fragger, Spectronaut, and DIA-NN requires implementation of scripts prior to proceeding with step 4 in Fig. 1. The input selection parameters for CURTAIN-PTM are selected through an inbuilt dropdown menu (step 4, Fig. 1) and summarized in Dataset S1. The visualization of experimentally determined PTM in each protein (step 10, Fig. 1) and comparison of experimental and database PTMs (step 11 and step 13, Fig. 1) are undertaken using Plotly (https://plot.ly) (45). For predictions of kinases that phosphorylate a phosphorylation site of interest (step 12, Fig. 1), we deployed the web API from the Kinase Library (https://kinase-library.phosphosite.org/site) (33). For selection of PTM databases (step 13, Fig. 1), we used PhosphoSitePlus (https://www.phosphosite.org/) (18), Protein Lysine Modification Database (PLMD) using downloaded data (19), CarbonylDB (http://carbonyldb.missouri.edu/CarbonylDB/index.php/) (20), GlyConnect (https://glyconnect.expasy.org/) (21), or Uniprot (https://www.uniprot.org/) (22). For PTM Motif analysis (step 9, Fig. 1), we used weblogo (https://logojs.wenglab.org/app/) (34). For multiple sequence alignments in CURTAIN-PTM, we used biomsalign (https://github.com/ppillot/biomsalign).

#### CURTAIN and CURTAIN-PTM Backend.

With CURTAIN and CURTAIN-PTM, the end user can save their working session (step 9 CURTAIN, Fig. 1, step 13 CURTAIN-PTM, Fig. 1) and receive a unique web link to retrieve and share the analysis containing both data and interactive plots. User sessional data are stored in JavaScript Object Notation (47) format using a backend built with the Django web framework (https://djangoproject.com) and Postgres database (https://www.postgresql.org). The main purpose of this backend is to provide permanent user storage as well as permission management for data access. Through a system controlled by the backend, if a user chooses to log in using their ORCID (https://orcid.org/) credential, the saved session can be bound to their account and they can control whether other users can access the session data.

#### Sample Preparation for Proteomic and Phosphoproteomic Analysis.

A549 PPM1H-BromoTag cells were cultured on 15-cm dishes (2 dishes per replicate). The cells were treated with 300 nM AGB1 or cis-AGB for 4-h or 24-h, were lysed in 600 μL of Lysis Buffer [50 mM Tris–HCl pH 7.5, 150 mM NaCl, 10% glycerol, 10 mM 2-glycerophosphate, 10 mM sodium pyrophosphate, 1 mM sodium orthovanadate, 1 μg/mL microcystin-LR, complete EDTA-free protease inhibitor cocktail (Roche), and 1% (v/v) Triton X-100] as described previously (23), following protein estimation, 3mg of lysate was processed using S-Trap assisted on-column digestion and eluted peptides were further purified using Sep-Pak cartridges. Ten percent of the eluate was taken for total proteomic analysis, and the remainder was subjected for phosphopeptide enrichment. A detailed protocol containing this step is available on Protocols.io (dx.doi.org/10.17504/protocols.io.261ged49yv47/v1).

#### TiO_2_-based Phosphopeptide Enrichment and Tandem Mass Tags Labeling.

Phospho-peptide enrichment was carried out using the High-Select TiO_2_ Phospho-peptide Enrichment kit (Thermo Fisher, A32993), as per the manufacturer’s instructions. A detailed protocol containing this step for phosphopeptide enrichment, TMT labeling, and High-pH fractionation analysis is available on Protocols.io (dx.doi.org/10.17504/protocols.io.261ged49yv47/v1).

#### LC-MS/MS Analysis and Database Searches.

Phosphopeptide fractions were analyzed using the Orbitrap Lumos Tribrid mass spectrometer in a DDA MS2 mode, and total proteome peptides were analyzed in a DDA-MS3 mode. For Fragpipe database search, the MS raw data were converted to mzML using the MS-convert tool (48) A detailed protocol containing this step is available on Protocols.io (dx.doi.org/10.17504/protocols.io.261ged49yv47/v1).

## Supplementary Material

Appendix 01 (PDF)

Dataset S01 (XLSX)

Dataset S02 (TXT)

Dataset S03 (XLSX)

Dataset S04 (XLSX)

Dataset S05 (XLSX)

Dataset S06 (XLSX)

Dataset S07 (XLSX)

Code S01 (ZIP)

Code S02 (ZIP)

Code S03 (ZIP)

Code S04 (ZIP)

Code S05 (ZIP)

Code S06 (ZIP)

Code S07 (ZIP)

## Data Availability

All the primary data that are presented in this study have been deposited in publicly accessible repositories. The CURTAIN and CURTAIN-PTM tools, CURTAIN utility, scripts for custom PTM database build as well as the primary immunoblotting data and statistical analysis have been deposited in Zenodo and provided as SI source code (https://doi.org/10.5281/zenodo.10079193, https://doi.org/10.5281/zenodo.10079194, https://doi.org/10.5281/zenodo.​10079341, https://doi.org/10.5281/zenodo.10079204, https://doi.org/10.5281/zenodo.10079224, https://doi.org/10.5281/zenodo.10079222, and https://doi.org/10.5281/zenodo.10079201) Proteomic data have been deposited in the ProteomeXchange PRIDE repository (Identifier: PXD043806) (49). All plasmids and antibodies generated at the MRC Protein Phosphorylation and Ubiquitylation Unit at the University of Dundee can be requested through our website https://mrcppureagents.dundee.ac.uk/. Supplemental datasets have been deposited in Zenodo (https://doi.org/10.5281/zenodo.10419853) (50). For the purpose of open access, the authors have applied a CC BY public copyright license to all Author Accepted Manuscripts arising from this submission.
