# Supplementary material for: CURTAIN—A unique web-based tool for exploration and sharing of MS-based proteomics data
Source: Proc Natl Acad Sci U S A. 2024 Feb 7;121(7):e2312676121. doi: 10.1073/pnas.2312676121 (PMC10873628; doi:10.1073/pnas.2312676121)
Supplement: Supplementary file 1 — Appendix 01 (PDF) [file pnas.2312676121.sapp.pdf]

Supporting information for

CURTAIN – A Unique Web-based tool for exploration and sharing of MS-based proteomics data

Toan K. Phung[1,2*], Kerryn Berndsen[1,2], Rosamund Shastry[1,2], Tran L.C.H.B Phan[1], Miratul M. K. Muqit[1,2], Dario R. Alessi[1,2*], Raja S. Nirujogi[1,2*]

[1] Medical Research Council (MRC) Protein Phosphorylation and Ubiquitylation Unit, School of Life Sciences, University of Dundee, Dow Street, Dundee DD1 5EH, U.K.

[2] Aligning Science Across Parkinson's (ASAP) Collaborative Research Network, Chevy Chase, MD, 20815, USA.

[#] Corresponding authors: T.P. tphung001@dundee.ac.uk; D.R.A. d.r.alessi@dundee.ac.uk; R.S.N. r.nirujogi@dundee.ac.uk

This PDF file contains:

Detailed materials and methods

Supplementary figures: S1 to S11

Legends for datasets: S1 to S7

Materials and methods:

Table 1. List of primary antibodies employed in this study.

Protein	Company	Catalog number	RRID number	Antibody species	Dilution Used
GAPDH	**Santa Cruz**	**sc-32233**	**AB_627679**	**Mouse**	**IB (1:2000)**
LRRK2 Cterm	**NeuroMab**	**N4241A/34**	**AB_2877351**	**Mouse**	**IB (1:1000)**
LRRK2 pSer935	**MRC PPU Reagents and Services**	**UDD2**	**AB_2921228**	**Rabbit**	**IB (1:1000)**

PPM1H (1-514)	MRC PPU Reagents and Services	DA018	AB_2923281	Sheep	IB (1 µg/ml)
PPM1H	Abcam	ab303536	AB_2941812	Rabbit	IB (1 µg/ml)
Rab10 pThr73	Abcam	ab230261	AB_2811274	Rabbit	IB (1:1000)
Rab10 total	nanoTools	0680-100/Rab10-605B11	AB_2921226	Mouse	IB (1:500)
PPM1H (CRISPR)	DU64668	PPM1H C-term KI BD-TAG-IRES2-GFP Sense guide A			pBabeD
PPM1H (CRISPR)	DU64674	PPM1H C-term KI BD-TAG-IRES2-GFP Antisense guide A			pBabeD
PPM1H (CRISPR)	DU69480	PPM1H C-term KI BD-TAG-IRES2-GFP Donor			pMK-RQ

Generation of A549 PPM1H-BromoTag CRISPR/CAS9 knock-in cell line

CRISPR was performed using a paired nickase approach to generate A549 PPM1H-BromoTag knock-in cell line (RRID number: CVCL_C8YV). Complementary oligos for the optimal guide pair A (a sense guide 5‘-GAAATGGCCCAGGGGATTGGG and an anti-sense guide 5’- GAGCTTGTTTCCATGTATTAA) were designed to target the C-terminus of PPM1H locus (ENSG00000111110). The sense guide was cloned into the pBabeD P U6 plasmid, and the anti-sense guide was cloned into the pX335 plasmid. The donor DNA containing IRES2 GFP for cell sorting purpose, was cloned into the pMK-RQ plasmid. CRISPR was done by co-transfecting wild-type A549 cells (80 % confluency, 6-well plates) with 500 ng of donor plasmid and 250 ng of each sense and antisense plasmids using Lipofectamine LTX according to the manufacturer’s instructions (Life Technologies). The transfected cells were grown for 24 h before selection with fresh DMEM media supplemented with 3 µg/ml puromycin for another 48 h. Single cells with positive GFP signal were sorted, then grown in individual wells of a 96-well plate for approximately 2 weeks. After reaching around 80 % confluency, individual clones were transferred into 6-well plates and homozygous A549 PPM1H-BromoTag clones determined by immunoblotting and genomic sequencing. A

detailed protocol in generating this cell line is available on Protocols.iO (dx.doi.org/10.17504/protocols.io.4r3l2268pl1y/v1)

MS Sample Processing

A549 PPM1H-BromoTag cells were cultured on 15 cm dishes (2 dishes per replicate). Cells were treated with 300 nM AGB1 or cis-AGB for 4 h or 24 h, from a 1000x stock made up in DMSO. Cells were lysed in 600 µl of Lysis Buffer [50 mM Tris–HCl pH 7.5, 150 mM NaCl, 10% glycerol, 10 mM 2-glycerophosphate, 10mM sodium pyrophosphate, 1 mM sodium orthovanadate, 1 µg/ml microcystin-LR, complete EDTA-free protease inhibitor cocktail (Roche) and 1% (v/v) Triton X-100] as described previously {Berndsen, 2019 #5853}, and lysates from the 2 dishes if cells pooled in 2 ml low-binding Eppendorf tubes. A small aliquot was taken for immunoblotting and the remainder of the sample was supplemented with 2% (w/v) SDS and subjected to sonication using a probe sonicator (BRANSON) employing 3 pulses. Samples were clarified at 17,000 x *g* at room temperature for 20 min and protein concentration measured using the BCA (bicinchoninic) protein assay {Walker, 1994 #6213}.

MS Sample preparation for total and Phosphoproteomic analysis

3 mg of SDS lysate was allotted for each sample and subjected to S-Trap assisted on-column digestion. The lysate from each sample was reduced by making up to a final 10 mM TCEP (tris(2-carboxyethyl)phosphine) from a 0.1M stock in 300 mM TEAB buffer (Triethylammonium bicarbonate, pH 8.0) and incubated on a Thermomixer at 60°C for 30 min with an agitation set at 1200 rpm. Samples were then brought to room temperature and alkylated by adding 40 mM Iodoacetamide (IAA) and further incubated on a Thermomixer at room temperature for 30 min with an agitation set at 1200 rpm and then quenched by adding additional further 5 mM TCEP and incubated at room temperature for 10 min. From a stock of 20% (w/v) SDS prepared in milliQ water, SDS concentration of each sample was increased to 5% (w/v) and acidified by bringing the final concentration to 1.2% (v/v) phosphoric acid from a 12% stock prepared in milliQ water. Immediately, 7 times the volume of lysate, S-Trap buffer (90% methanol (vol/vol) in 100 mM TEAB buffer) was added, and the resulting solution transferred to a S-Trap midi column. The columns were centrifuged at room temperature at 2000 rpm for 1 min. Columns were washed by adding 4 ml of S-Trap buffer and centrifuged at 2000 rpm for 1 min and this step was repeated four times. Flowthrough was discarded at each step and after the final wash, the columns were centrifuged at 3000 rpm for 1 min and the columns were transferred to a new 15 ml collection tubes. For each S-Trap column, 30 µg (1 to 100 ratio of protease to cell extract amount) of a mixture of Trypsin+Lys-C (Thermo A41007) dissolved in 350 µl of 50 mM TEAB and was added. In addition, 300 µg of Tosyl phenylalanyl chloromethyl ketone (TPCK) treated trypsin (Sigma,4352157) was dissolved in 350 µl of 50 mM TEAB buffer and added to the S-TRAP column. Columns were immediately centrifuged at 100 rpm for 1 min to remove air-bubbles and the flowthrough was re applied to the column which were then placed on a Thermo mixer with a 15 ml rack and incubated at 47 ^{o}C for 90 min without agitation. The temperature was reduced to room temperature and digestion was continued the on-column digestion overnight (~ 16 h). 500 µl of 50 mM TEAB was then added and column centrifuged 100 rpm for 1 min. Next, 500 µl of 0.1% (v/v) formic acid in Milli-Q water was added and centrifuged 100 rpm for 1 min. 500 µl of 80 % (v/v) Acetonitrile in 0.1% formic acid (v/v) was added and centrifuged at 100 rpm for 1 min and this step two further times. Eluates were then vortexed and centrifuged at 3,000 rpm for 10 min to precipitate any

debris or undigested protein amounts. Eluates were then transferred to 2 ml low binding Eppendorf tubes, placed on dry ice for 10 min and vacuum dried using a speedvac concentrator and if required stored in -80 freezer.

The dried samples were resuspended in 500 µl 1% (v/v) TFA (trifluoroacetic acid) and incubated for 30 min on a thermomixer at 1800 rpm at room temperature, sonicated on a water bath sonicator for 10 min and centrifuged at 17,000 x *g* for 10 min at room temperature. The supernatant was transferred to new 2 ml low binding Eppendorf tubes and pH was checked by pipetting 0.5 µl onto the pH strip to ensure samples were pH 2.0. Sep-Pak cartridges (50 mg tC18) were placed into 15 ml falcon tubes. The cartridges were activated by adding 1 ml of 100% acetonitrile and centrifuged at 100 x *g* for 1 min and this step repeated a further 3 times. Cartridges were then equilibrated by undertaking 4 x 1 ml washes with 0.1% (v/v) TFA. Dissolved peptide samples were loaded onto the equilibrated cartridges and allowed to pass through by gravity (takes 15-30 min) and the flowthrough was reapplied. The cartridge was washed four times in 1 ml 0.15% (v/v) formic acid and flowthrough disregarded. Peptides were eluted using 3 washes of 300 µl of 50% acetonitrile in 0.15% (v/v) formic acid. 1 µl aliquots were taken for a digestion check and 10% (90 µl) of each sample was taken for total proteomic analysis. The remaining samples were snap-frozen on dry ice, vacuum dried and stored at -80 °C. A detailed protocol containing this step for combined total and phosphoproteomic analysis is available on Protocols.io (dx.doi.org/10.17504/protocols.io.261ged49yv47/v1).

TiO2-based Phosphopeptide enrichment

Phospho-peptide enrichment was carried out using the High-Select TiO_2 Phospho-peptide Enrichment kit (Thermo Fisher, A32993), as per manufacturer's instructions. Lyophilized samples were resuspended in 150 µl of "Binding/Equilibration buffer" (provided with the kit) and incubated at room temperature on a Thermomixer at 1800 rpm for 30 min. Samples were sonicated in a water bath sonicator for 10 min and centrifuged at 17,000 x *g* for a further 10 min. The supernatant was transferred to a protein low-binding 1.5 ml Eppendorf tube and pH was checked by pipetting 0.5 µl onto the pH strip to ensure samples were <pH 3.0. TiO_2 spin tip (provided in kit) was placed into a 2 ml low binding Eppendorf tube. Tips were washed using 20 µl wash buffer (provided in kit) and centrifuged at 3000 x *g* for 2 min, and equilibrated in 20 µl Binding/Equilibration buffer and centrifuged for a further 2 min. The equilibrated tip was transferred to a new 2 ml protein low-binding Eppendorf tube. The resuspended samples were loaded onto the tip and centrifuged at 1000 x *g* for 5 min. The flowthrough was re-applied, and centrifugation repeated. The tips were transferred to a new 2 ml protein low-binding tube, washed with 20 µl Binding/Equilibration column, followed by 20 µl wash buffer, centrifuging at 3000 x *g* for 2 min each time. Both wash steps were repeated one more time and washed with 20 µl of LC-MS grade water. The tips were placed in a new 1.5 ml protein low-binding Eppendorf tube and the phospho-peptides eluted using 50 µl Elution buffer (provided with kit) and centrifugation at 1000 x *g* for 5 min. The elution step was repeated one more time. 2 µl was taken for a phospho-enrichment check. The remaining eluate was snap-frozen on dry ice and vacuum dried. Samples were then subjected to C18 clean-up using 50 mg 1cc tC18 Sep-Pak cartridges, as described above. A detailed protocol containing this step for combined total and phosphoproteomic analysis is available on Protocols.io (dx.doi.org/10.17504/protocols.io.261ged49yv47/v1).

TMT labeling

The Phospho-enriched as well as the total proteomic samples were resuspended in 30 µl fresh 50 mM TEAB and incubated at room temperature on a thermomixer at 1800 rpm for 1 hr. Samples were centrifuged at 17,000 x *g* for 3 min and supernatant transferred to 0.5 ml protein low binding Eppendorf tube. 0.5 mg of each TMT reagent (Thermo Fisher, A44520) was resuspended in 100 µl 30% anhydrous acetonitrile to a final concentration of 5 µg/µl. TMT labels were added to the samples (10 µl for phospho-proteomics, and 20 µl for total proteomics), incubated at room temperature on a thermomixer at 1200 rpm for 2 h and 50 mM TEAB added to adjust the total volume to 100 µl. The mixtures were incubated on a thermomixer at 1200 rpm for a further 10 min at room temperature. A total of 2 µl aliquots of each sample were removed to verify the TMT labeling efficiency. Furthermore, 2 µl of each sample was removed and pooled to prepare a mini pool to verify the absolute peptide abundance in each sample. Once label checks were complete, samples were quenched by adding 3 µl of 5% hydroxyl amine and incubating at room temperature on a thermomixer at 1250 rpm for 20 min. Samples were then pooled appropriately to achieve equal abundance of each TMT labeled peptides. We have generated a simple web-based tool to determine the specific volumes of each sample that need to be mixed to equalize the peptide levels (https://samplepooler.proteo.info). The final mixture was centrifuged at 17,000 rpm for 1 min and, snap-frozen on dry ice and vacuum dried. A detailed protocol containing this step for combined total and Phosphoproteomic analysis is available on Protocols.io (dx.doi.org/10.17504/protocols.io.261ged49yv47/v1).

High-pH reversed-phase liquid chromatography fractionation.

Total proteome and Phosphoproteome samples were next fractionated using high-pH reversed-phase liquid chromatography fractionation. Dried samples were resuspended in 110 µl Solvent A [10mM ammonium formate in LC-MS grade water, pH adjusted to 10.0 using MS grade ammonium hydroxide] and incubated at room temperature on a thermomixer at 1800 rpm for 20 min. 100 µl of the phospho-proteomic and total proteome sample was transferred to LC vials for high pH fractionation using XBridge 25 cm C18 column (Waters, 186003010) on a Dionex 3000 LC system. The column was equilibrated at 3% solvent B (10 mM ammonium formate in 80% acetonitrile pH 10) at 0.275 ml/min flow rate. The sample was loaded, and the column washed with 3% solvent B for 20 min to remove excess unlabeled TMT reagent. A linear gradient from 3 to 40% solvent over 90 min was applied and 96 fractions were collected in a 96 deep well plate. Fractions were concatenated into 48 protein low binding 1.5 ml Eppendorf tubes (dx.doi.org/10.17504/protocols.io.bs3tngnn), snap-frozen, vacuum dried and stored at -80 °C.

Dried fractions were resuspended in 50 µl LC buffer [3% acetonitrile in 0.5% Formic acid] and incubated on a thermomixer at 1800 rpm for 1 h at room temperature. Samples were vortexed and centrifuged at 17,000 x *g* for 5 min. 10 µl of each sample was transferred to LC vials and subjected to mass spectrometry. A detailed protocol containing this step for combined total and Phosphoproteomic analysis is available on Protocols.io (dx.doi.org/10.17504/protocols.io.261ged49yv47/v1).

MS data acquisition and database searches

Phosphopeptides were analyzed on a Orbitrap Lumos Tribrid mass spectrometer in-line with Dionex RSLC 3000 nano-liquid chromatography system. Peptides were loaded on a 2cm trap column at 5 µl/min flow rate and resolved on a 50 cm analytical column at 350 nl/min

flow rate. Data was acquired using Data dependent acquisition in MS2 mode. Full MS was acquired in the mass range of 350 - 1500 m/z and measured using Orbitrap mass analyzer at a resolution of 120,000 at m/z 200. MS2 scans were acquired in a top 15 data dependent mode and fragmented using a normalized higher energy collisional dissociation (HCD) 37.5% and measured using Orbitrap mass analyzer at a resolution of 45,000 at m/z 200. Quadrupole mass filter was set to 0.7 Da for MS2 scans. AGC targets and Ion injection times for both MS1 and MS2 were set at 3E6 and 2E5 for 30 ms and 120 ms respectively. Total proteome was analyzed using the same MS instrument and columns except the data was acquired using SPS-MS3 mode. Full MS1 was acquired using an Orbitrap mass analyzer at a resolution of 120,000 at m/z 200. MS scans were acquired at a top speed for 2 sec and fragmented using 32% HCD and measured using ion trap mass analyzer. Synchronous precursor selection (SPS) for MS3 was performed using 10 MS2 fragment ions and further fragmented using normalized 65% HCD energy and measured using Orbitrap mass analyzer at a resolution of 60,000 at m/z 200. A detailed protocol containing this step for combined total and Phosphoproteomic analysis is available on Protocols.io (dx.doi.org/10.17504/protocols.io.261ged49yv47/v1).

Total proteome raw data searched using FragPipe version 18.0. In-built TMT-Pro16 plex workflow was selected as default settings and searched data using MS-Fragger version 3.5 against the Human Uniprot database (July 2022: UP000005640). Oxidation of Met, Phosphorylation of STY, Deamidation of NQ were selected as variable modifications and Carbamidomethylation of Cys as fixed modifications. Trypsin as protease with two missed cleavages were allowed. Phosphoproteomics data was searched using MaxQuant version 2.0.3.0 and searched against the Human Uniprot database (May 2021). TMT-Pro 16plex workflow was selected with 0.75 Precursor ion filter (PIF) was sent as a filter. Trypsin was selected as protease with a maximum of two missed cleavages were allowed. Oxidation (M); Acetyl (Protein N-term); Deamidation (NQ) and Phosphorylation of (STY) were set as variable modifications and Carbamidomethylation of Cys as fixed modification. 1% False discovery rate for PSM, sites and protein level were applied. The search output files from total proteome and Phosphoproteomics data was further processed using Perseus version (1.6.15.0) for statistical analysis and further data was visualized using CURTAIN and CURTAIN-PTM.

CURTAIN and CURTAIN-PTM backend

We designed CURTAIN and CURTAIN-PTM so that the end user can save their session (Step 14, Fig 1) and generate a unique web link to retrieve and share the analysis of data and plots, which can also be published (Step 15, Fig1). If the user has logged into CURTAIN or CURTAIN-PTM employing an ORCID (https://orcid.org/), this associates all data generated by that user and they will be able to view and track all saved sessions associated across different projects within an ownership table (Step 16, Fig 1). By default, when a user has logged in using an ORCID, the data is saved as private. For data to be viewed and shared by others with a weblink, the setting needs to be manually changed by the owner to “Share” (Step 16, Fig 1). If the user does not login with a user ID, then all data can be viewed and shared with anyone through the saved web link.

Within the backend, the user information table contains all the basic non-session related data such as username and password. When the user chooses to save their working session, the frontend will package the current input data, plot settings and user data selections into a single JavaScript Object Notation (JSON) and send the data to the backend. The overall flow of data is summarized in (SFig 1A). The backend was developed using the Python-based Django web framework (https://djangoproject.com) to receive and

store user data within a Postgres (https://www.postgresql.org) database with a general schema as represented in SFigure 1B. The schema includes five separate tables namely, user information, session ownership, session metadata, session access token, and user data filter list (SFig 1B).
Upon receiving the uploaded session packages, the data is saved into the session metadata table with a unique random string as weblink id. This table contains information on the date the session has been created, unique id, private or share status, and type of CURTAIN session (i.e., total proteomics or PTM). For users' logging in using ORCID, the backend automatically creates an account using the ORCID as username and an encrypted randomized password.

Session data retrieval
When viewed through the web browser, CURTAIN first verifies whether the session is marked as "shared" and permission denied if the session is marked "private". With successful handshake, CURTAIN then requests the associated session data from the backend which is unpacked and processed with the original settings that were saved by the owner. The data that can be viewed and reanalyzed. The new session can be saved as needed and this will generate a new session link rather than overwrite the previous sessions.

CURTAIN and CURTAIN-PTM backend host server
CURTAIN and CURTAIN-PTM are currently hosted on a dedicated web server, using the Debian 11 operating system. Each component of the backend including SQL database and Django instance, are operated within their own docker container. The frontends are hosted within GitHub Page. We have also provided instructions for self-hosting the frontends using docker and nginx. All the necessary instructions for setting up CURTAIN can be found at https://github.com/noatgnu/CURTAIN.
Note that for the Phosphositeplus PTM database that can be optionally selected to compare experimental data within CURTAIN-PTM (Step 11, Fig 1B) requires license compliance for non-academic users'. The minimum recommended hardware requirements for CURTAIN or CURTAIN-PTM is 4 GB of RAM, and 1 TB of SATA HDD allowing groups to self-host.

Code availability:
CURTAIN was created using the Angular Web framework (https://angular.io/) and Python. The code is freely available on Github for CURTAIN (accession number https://doi.org/10.5281/zenodo.10079193, Source code 1), CURTAIN-PTM (accession number https://doi.org/10.5281/zenodo.10079194, Source code 2) and associated components, namely backend (accession number https://doi.org/10.5281/zenodo.10079341, Source code 3), UniprotParserjs (accession number https://doi.org/10.5281/zenodo.10079222, Source code 4), common access JavaScript Application Protocol Interface (API) allowing others to link to and interact with CURTAIN backend (accession number https://doi.org/10.5281/zenodo.10079204, Source code 5), and Python API (accession number https://doi.org/10.5281/zenodo.10079224, Source code 6) via the MIT open-source license (https://opensource.org/license/mit/). CURTAIN is designed to run on all commonly used web browsers and has been tested on Safari, Chrome, Microsoft Edge and Firefox.

Video Tutorials and Google Group:
Videos were recorded and edited using the screen-capture recording software Camtasia (https://www.techsmith.com/video-editor.html). Six videos were created in total and these have been uploaded and organized into two separate playlists on our YouTube channel (https://www.youtube.com/@CURTAIN-me6hl), which is linked to a Google Business account set up exclusively for this project.
The first playlist, "Curtain Tutorials" (https://www.youtube.com/watch?v=6YCBrJrYCF4&list=PLFXgFou6OKWBAVcKAEpNA_nVopBRAIHoh), contains a series of four videos, set up to be played in the following order: "How to Upload your Data into Curtain" (https://www.youtube.com/watch?v=6YCBrJrYCF4), "How to Customize and Export Plots in Curtain" (https://www.youtube.com/watch?v=jymRzfZWzmY), "How to Evaluate Data Quality in Curtain" (https://www.youtube.com/watch?v=LjAcNKyvA2I), and, finally, "How to Learn More about Structure, Interactors and Biology of Experimental Hits using Curtain" (https://www.youtube.com/watch?v=MnUelfbuz2E).
The second playlist, "CurtainPTM Tutorials" (https://www.youtube.com/watch?v=gHJIDDBwqcQ&list=PLFXgFou6OKWBnT8fx0C9-TwSepDM7Ivv0), contains two videos, the first entitled "How to upload your data into CurtainPTM" (https://www.youtube.com/watch?v=gHJIDDBwqcQ), and the second, "How to explore your data using CurtainPTM" (https://www.youtube.com/watch?v=fdM6EDgGAWM).
The video tutorials use the data described in the paper, so users can download this and follow the tutorials in a hands-on manner if they wish.
Users can subscribe to the channel and leave comments directly beneath the videos. We have also generated a Google group (https://groups.google.com/g/curtain-proteomics) to enable users to provide feedback and report bugs.

S-Fig1

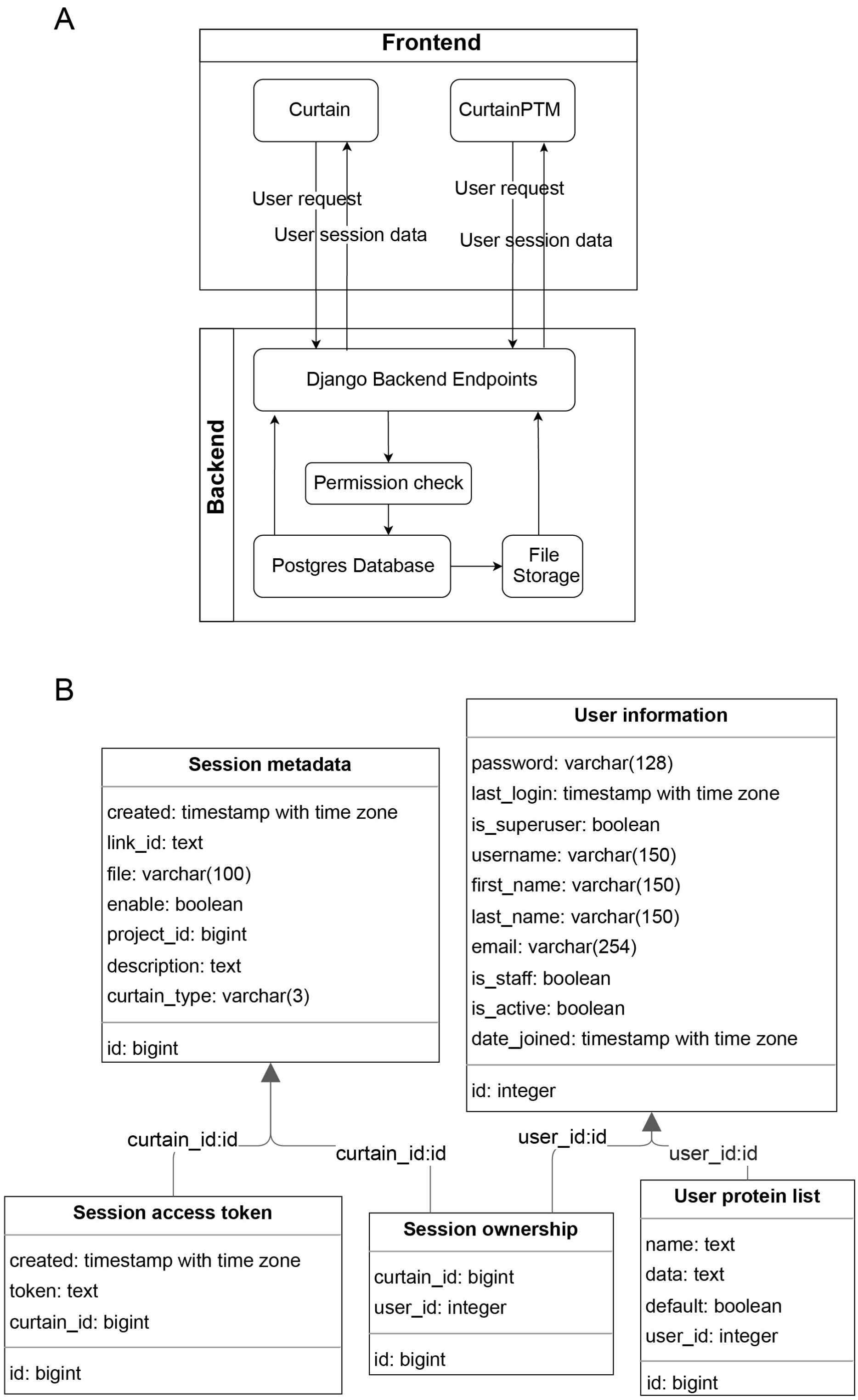

SFigure 1. **Architecture of CURTAIN and CURTAIN-PTM**. (A) CURTAIN and CURTAIN-PTM utilize the same backend. When a user request is accepted by the backend, a permission check is carried out to screen whether the request is sharable or private and can gain access to the database. (B) Summarizes the data structure within the database.

SFig: 2

A B

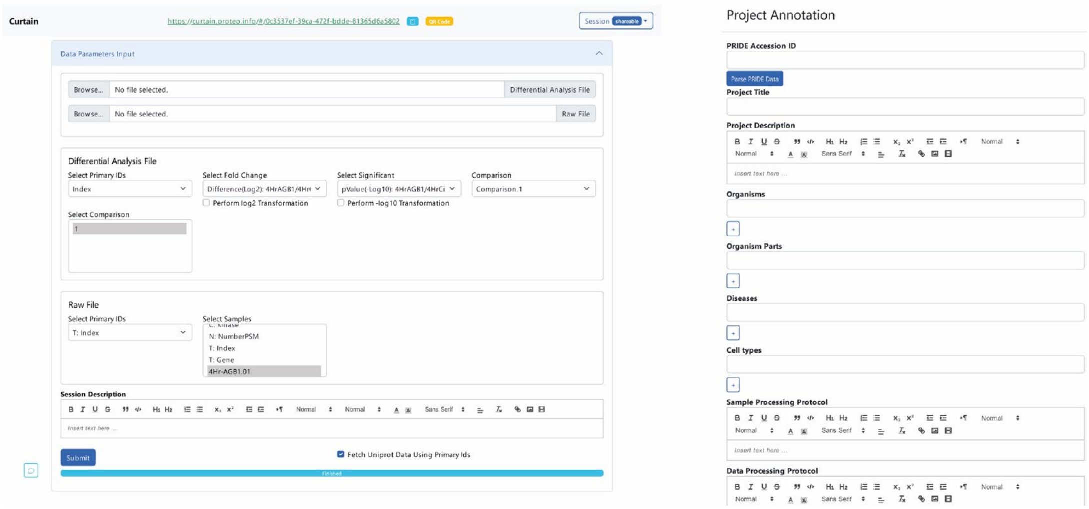

SFigure 2: **Overview of CURTAIN user interface**. (A) Screenshot of the base parameter input interface from a loaded CURTAIN session link. Within the base input, the user can import the two required differential analysis (Differential analysis file from the interface) and processed primary data files (Raw file from the interface). Further detail on selection parameters is described in Dataset S1. From the dropdown under “Session” button, the user can choose to include metadata for the project in Project Annotation (B). The input forms are designed to be similar to that of the data submission process for the PRIDE database.

SFig 3

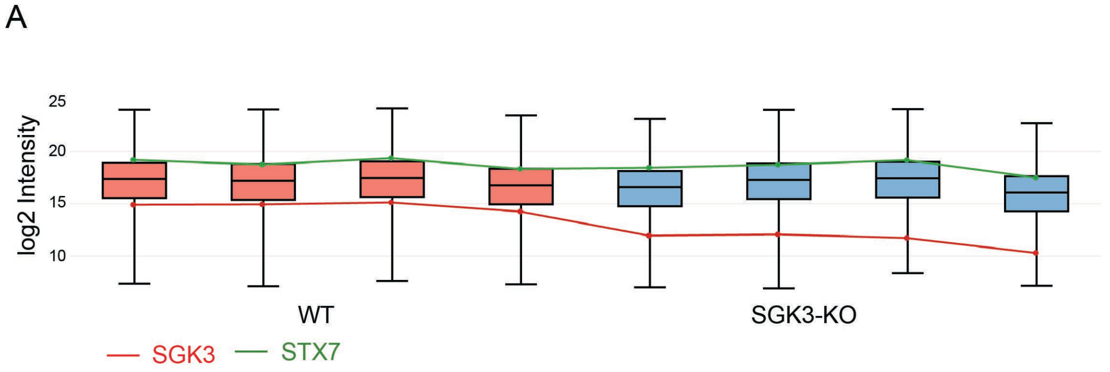

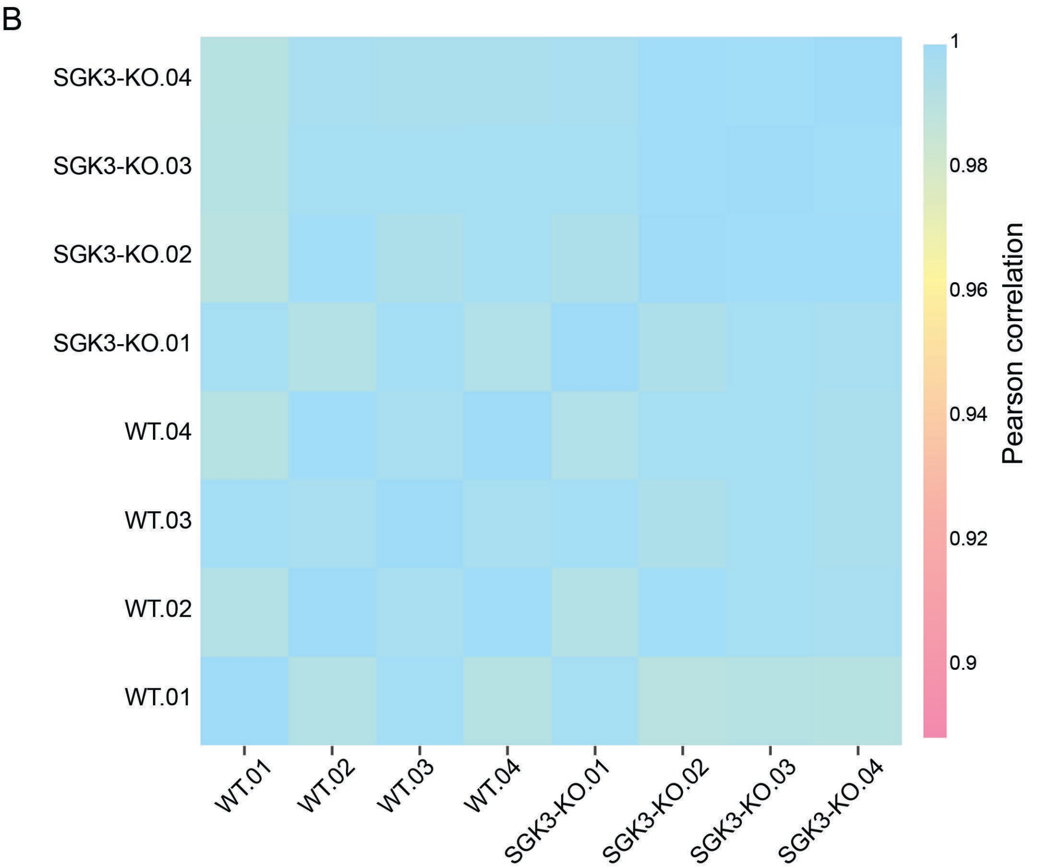

SFigure 3: **Overview of quality control visualization capabilities of CURTAIN using SGK3 experiment data described in Figure 3**. (A) Profile plot that is composed of box plots built from log2 of imported primary data depicting the data distribution and quality of the proteomics data. Within (A), we annotated the primary data from SGK3 and STX7 across all samples. (B) is a correlation matrix calculated within the browser from the same imported primary data. The color scale described the Pearson correlation value within the matrix. The Curtain link for this data is https://curtain.proteo.info/#/6ac93165-d351-4634-9683-ed342a6feaa8

SFig 4

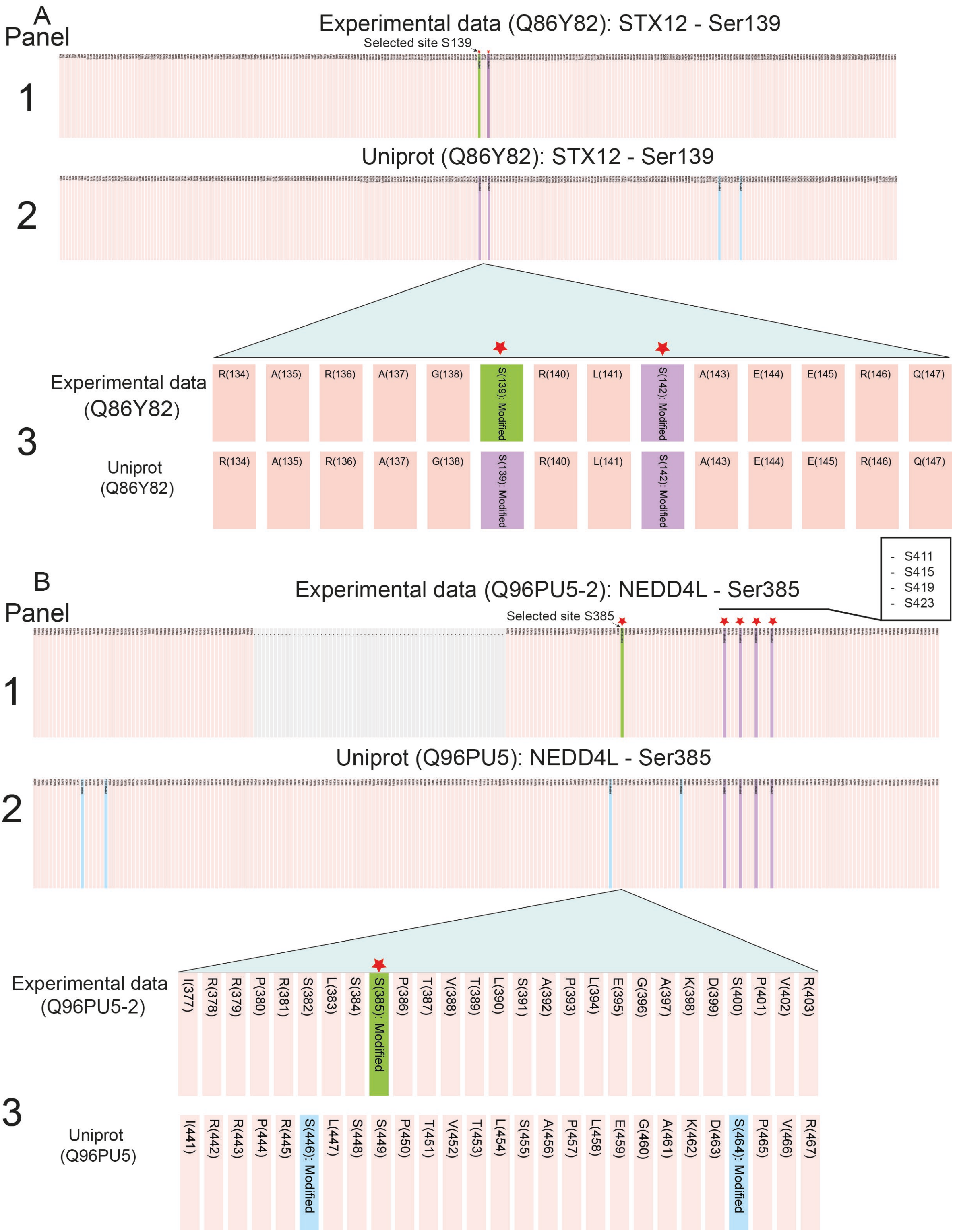

SFigure 4: **Visualization of PTM data on linear protein sequence**. From the analysis undertaken in Fig 4A, two hits namely STX12 and NEDD4L were selected for visualization of

PTM data on a linear protein sequence. Note that CURTAIN-PTM numbering of residues is based on “experimental data” provided by the MS search algorithm. which may differ from the canonical sequence displayed in selected databases or discussed in the literature. CURTAIN-PTM analysis of the phosphoproteomic data analyzed in Fig 4A, revealed that STX12 was phosphorylated at 2 sites (Ser139 and Ser142) whilst NEDD4L was phosphorylated at 5 sites (Ser385, Ser411, Ser415, Ser419 and Ser423). The CURTAIN-PTM outputs from these analyses are shown in Panel-1. Highlighted sites selected by the user are in green. Other identified non-selected sites are displayed in purple. CURTAIN-PTM automatically marks STX12-S139 (A) and NEDD4L sites (Ser385, Ser411, Ser415, Ser419 and Ser423) (B) with an asterisk as the levels of sites change significantly between experimental conditions (±IGF1). The other sites not marked with an asterisk do not change with experimental conditions and are not highlighted. Curtain permits experimental PTMs to be compared with publicly available databases such as Uniprot and the output of this comparison is displayed in Panel-2. Other phosphorylation sites that are listed in the database but not detected in the phospho-proteomic data are shown in blue. To better visualize sites of interest, CURTAIN-PTM permits to zoom-in within a selected area Panel-3. The upper panel 3 lists the sequence from the MS search algorithm whilst the lower panel lists the sequence based on the selected database. Note for STX12 (A) the numbering of residues is identical, as the same splice variant was analyzed in MaxQuant and Uniprot, however for NEDD4L (B) the residue numbers differ as Uniprot selected a different isoform to MaxQuant.

.

SFig 5

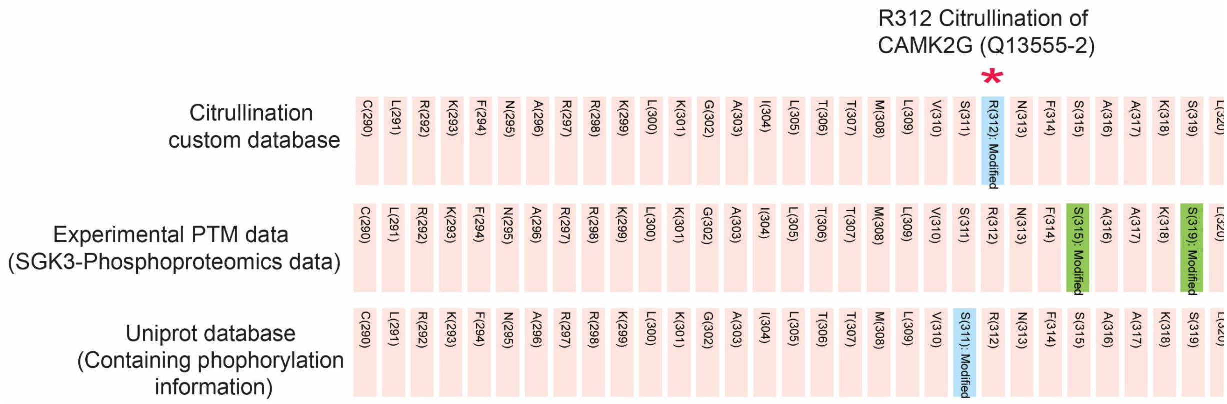

SFigure 5: Visualization of custom PTM database within CURTAIN-PTM: A custom PTM database can be imported within CURTAIN-PTM allowing users to use their own source of PTM metadata. An example citrullination PTM database was generated from a published study and then imported and visualized on a selected CAMK2G protein. Protein sequence alignment of CAMK2G (Q13555-2), top panel depicting the Citrullination at position R312 indicated with asterisk, middle and bottom panel depicting the experimental PTM data and Uniprot database. https://curtainptm.proteo.info/#/66b61571-8dce-4396-9680-11ce6bb1d24a

SFig 6

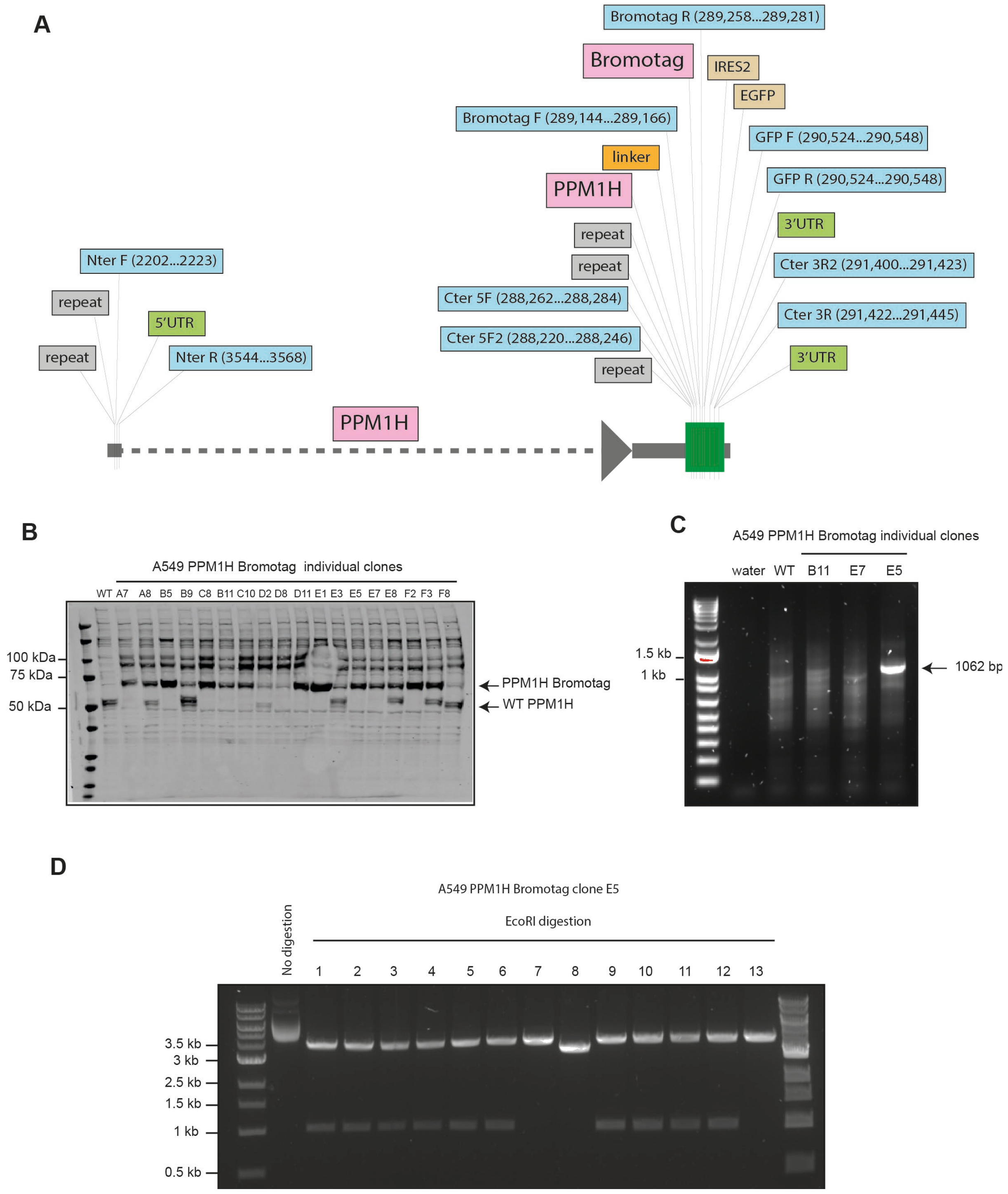

SFigure 6: **Verification of PPM1H-BromoTag knock-in cells**. (A) The map of PPM1H locus containing C-terminal BromoTag and IRES2 GFP. (B) Immunoblotting was performed to screen individual clones of PPM1H-BromoTag using anti-PPM1H antibody at concentration 1 µg/ml (sheep polyclonal antibody, MRC PPU Reagents and Services, DA018). Wild-type (WT)

A549 cells were included as a control. (C) PCR was performed to confirm the presence of PPM1H-BromoTag in potential homozygous clones observed from immunoblotting. A pair of primers (Cter 5F2: 5'- CTTGCTGAACTTACATTGGTCAAGAGG and BROMOTAG R: 5'-ACTTGATTGTGCTCATGTCCATGG) were used to amplify an amplicon with expected size at 1062 bp. Water and wild-type (WT) A549 were included as negative controls. (D) The clone E5 was selected to clone into the StrataClone PCR cloning vector pSC-B-amp/kan according to the manufacturer's instructions (Stratagene). Thirteen random positive colonies were digested with EcoRI restriction enzymes. All thirteen plasmids were sent for sequencing with M13 forward primer (5'- GTAAAACGACGGCCAGTG) and M13 reverse primer (5'-GGAAACAGCTATGACCATG). Plasmids 1, 2, 3, 4, 5, 6, 9, 10, 11, 12 showed sequences matching with the template of PPM1H-BromoTag. In contrast, the plasmids 7, 8, 13 did not show any match with the template, indicating that the fragments cloned into these plasmids are just non-specific PCR products. These results confirm that PPM1H-BromoTag clone E5 is a homozygous clone which was used for all experiments in this study.

SFig 7

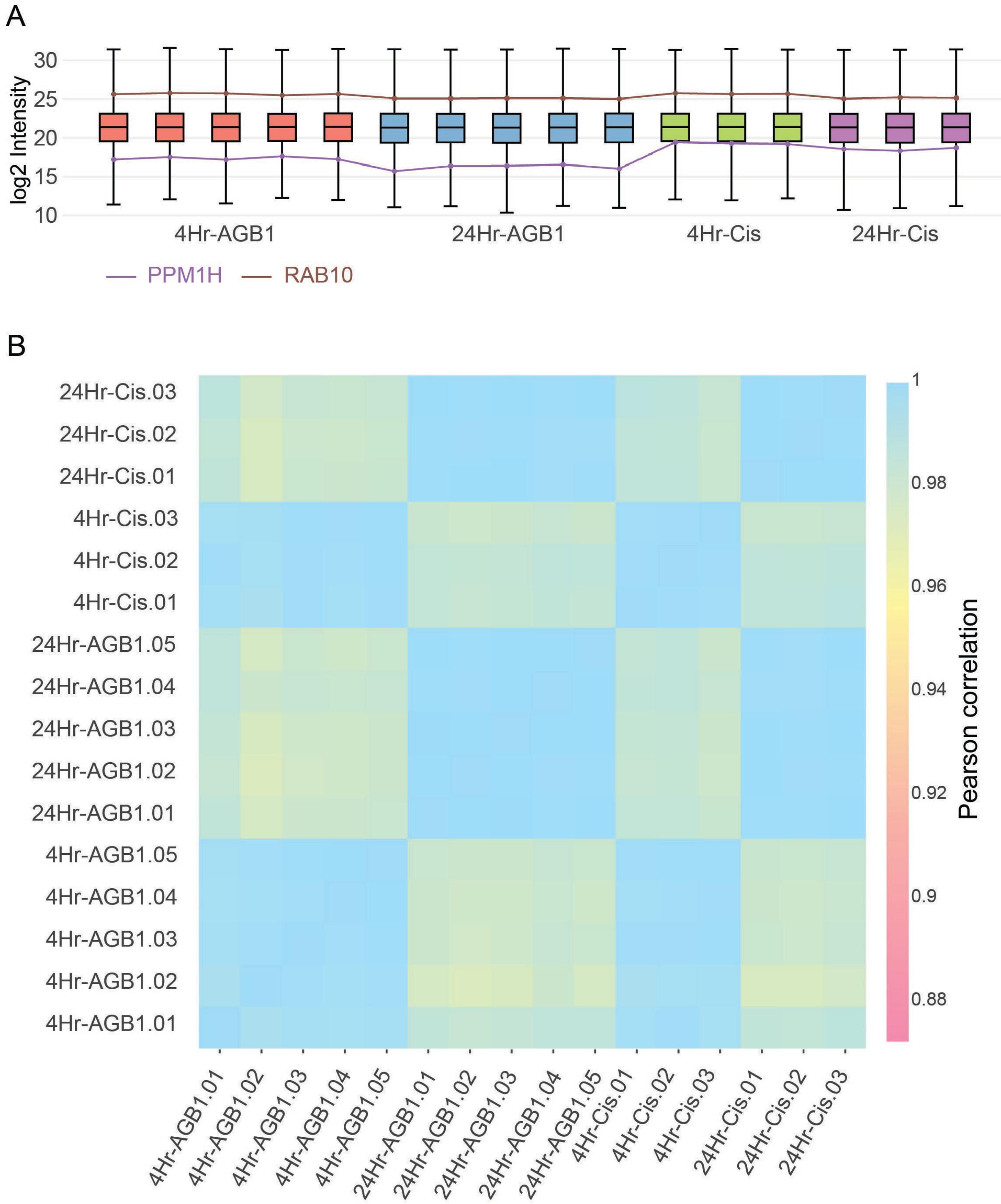

SFigure 7: Quality control visualization of PPM1H-BromTAG experimental data shown in Figure 7. (A) Profile plot that is composed of box plots built from log2 of imported primary data depicting the data distribution and quality of the proteomics data. Within (A) we annotated the primary data from total PPM1H and RAB10. (B) is a correlation matrix calculated from the same imported data. The matrix heat map showed that there are minor differences in correlation between 4h and 24h data. The Curtain link for this data is https://curtain.proteo.info/#/273b6d5e-2f21-43a6-a33c-e6ac53e801bd

SFig 8

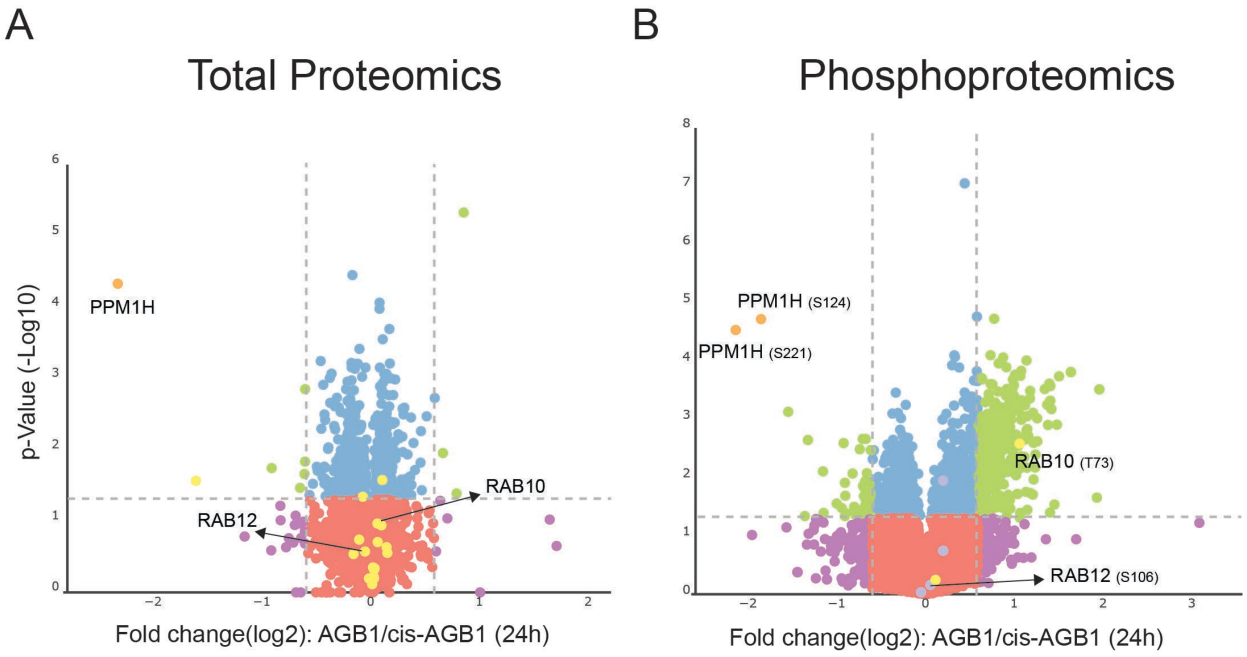

SFigure 8: **Visualization of PPM1H total and phosphoproteomics data using CURTAIN and CURTAIN-PTM**. The lysates produced from the experiments described in Fig 6B in which cells were treated for 24h with cis-AGB1 or AGB1 were subjected to total and phospho-proteomic analysis with data being analyzed with MS-Fragger and MaxQuant. CURTAIN generated volcano plot are presented for the total proteomic (A, https://curtain.proteo.info/#/244cc639-3f22-40e3-b528-564b4989d8f6) and Phospho-proteomic (B, https://curtainptm.proteo.info/#/5510ee39-5695-4995-a085-13adcc47e5cc) and proteins of interest highlighted in black. The same color coding is used as described in Fig 3A. C-E) The primary total and Phosphosite intensities of indicated hits were shown as bar graphs and violin plots.

SFig 9

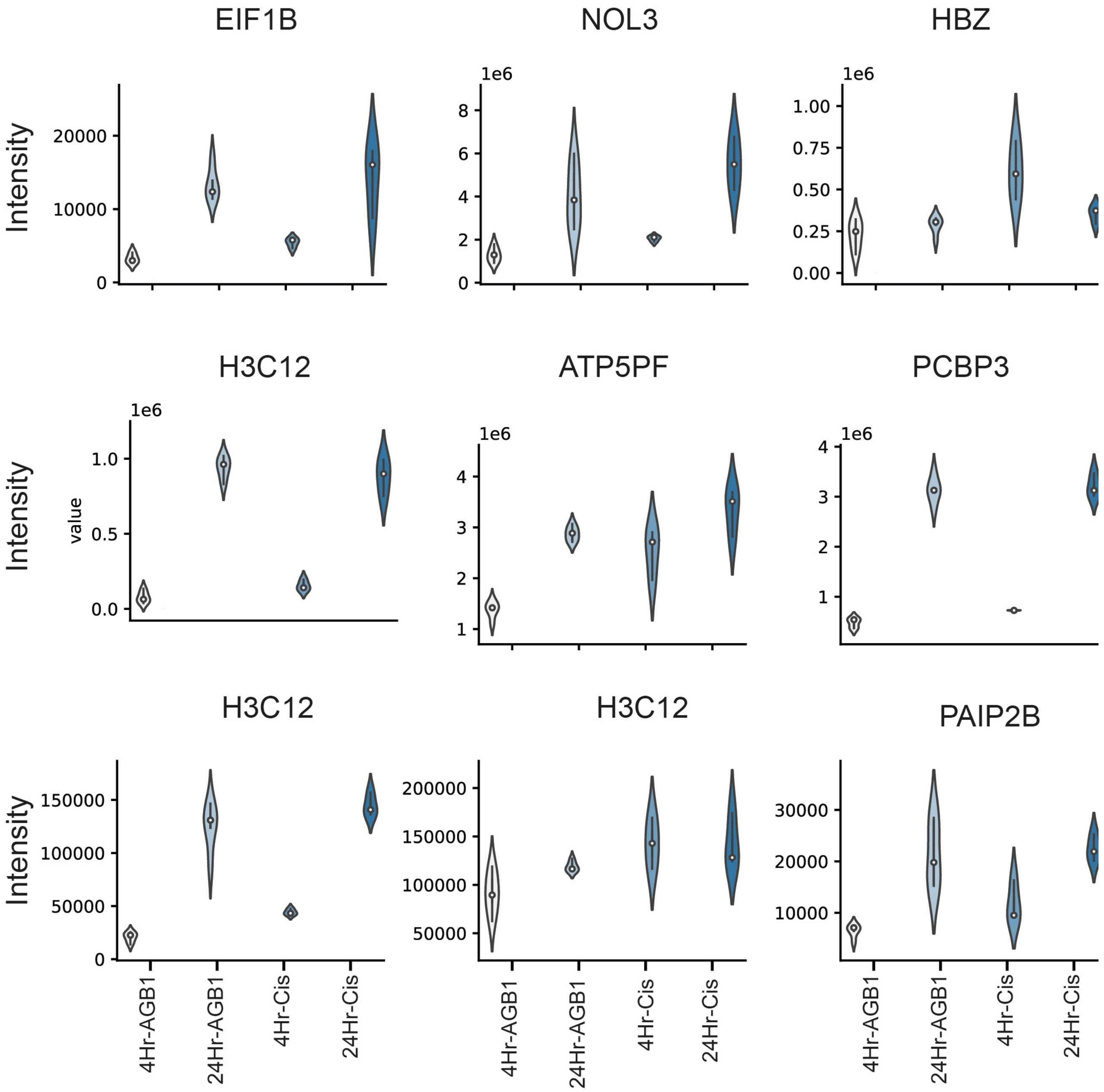

SFigure 9: Proteins whose levels are impacted following PPM1H-BromoTag depletion. A grid of violin plots of significantly changed proteins with log2 fold change greater than 1.0 derived from the experiments described in Figure 7A and SFigure 6A. The plots are composed from total proteomics data from both 4h and 24h samples.

SFig 10

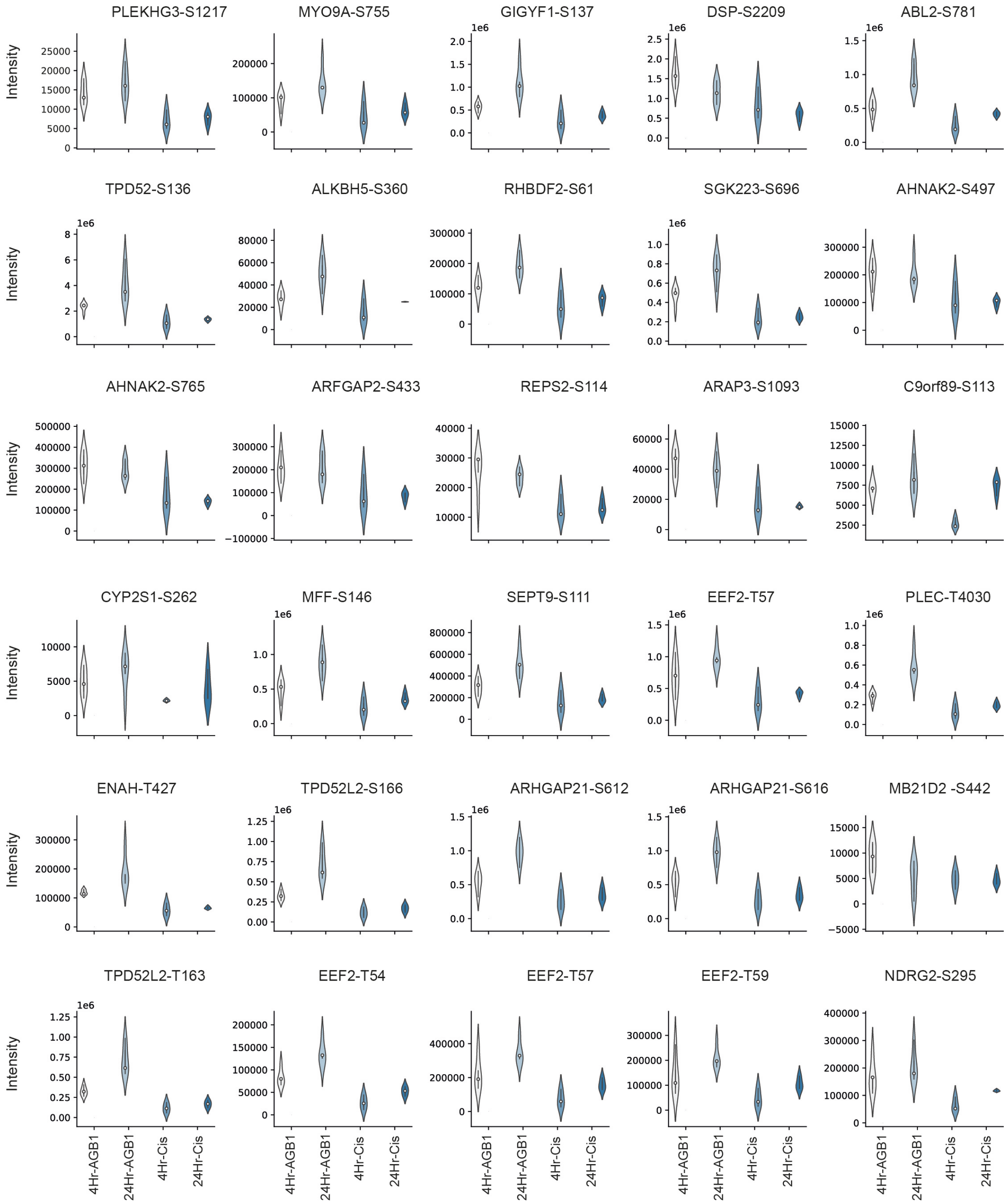

SFigure 10: **Phosphosites whose levels are impacted following depletion PPM1H-BromoTag**. A grid of violin plots of significantly changed proteins with log2 fold change greater than 1.0 derived from the experiments described in Figure 7B and SFigure 6B. The plots are composed from phospho-proteomics data from both 4h and 24h samples.

SFig 11A

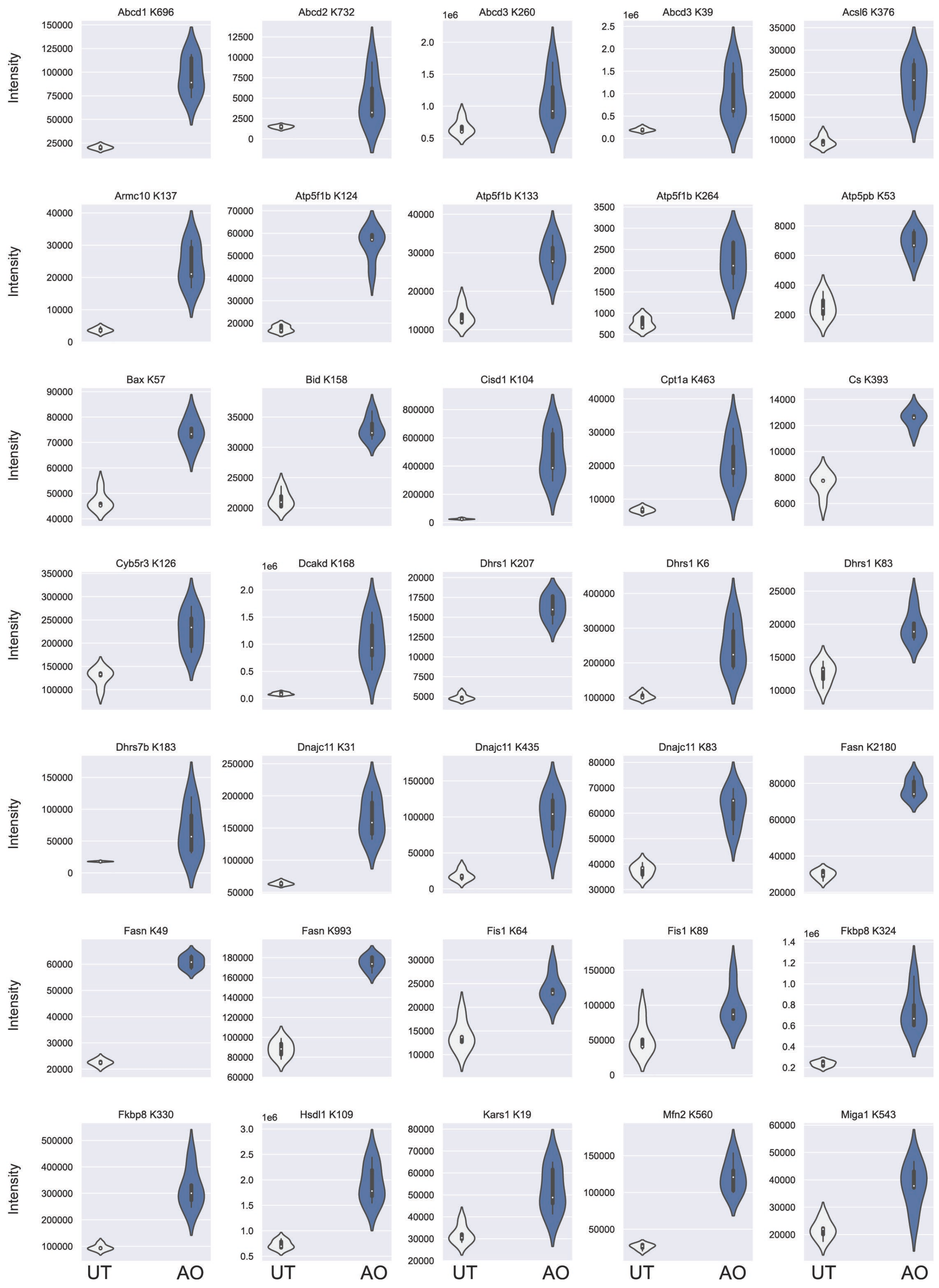

Abcd1 K696
Abcd2 K732
Abcd3 K260
Abcd3 K39
Acsl6 K376
Armc10 K137
Atp5f1b K124
Atp5f1b K133
Atp5f1b K264
Atp5pb K53
Bax K57
Bid K158
Cisd1 K104
Cpt1a K463
Cs K393
Cyb5r3 K126
Dcakd K168
Dhrs1 K207
Dhrs1 K6
Dhrs1 K83
Dhrs7b K183
Dnajc11 K31
Dnajc11 K435
Dnajc11 K83
Fasn K2180
Fasn K49
Fasn K993
Fis1 K64
Fis1 K89
Fkbp8 K324
Fkbp8 K330
Hsdl1 K109
Kars1 K19
Mfn2 K560
Miga1 K543
Intensity
1e6
UT
AO

SFig 11B

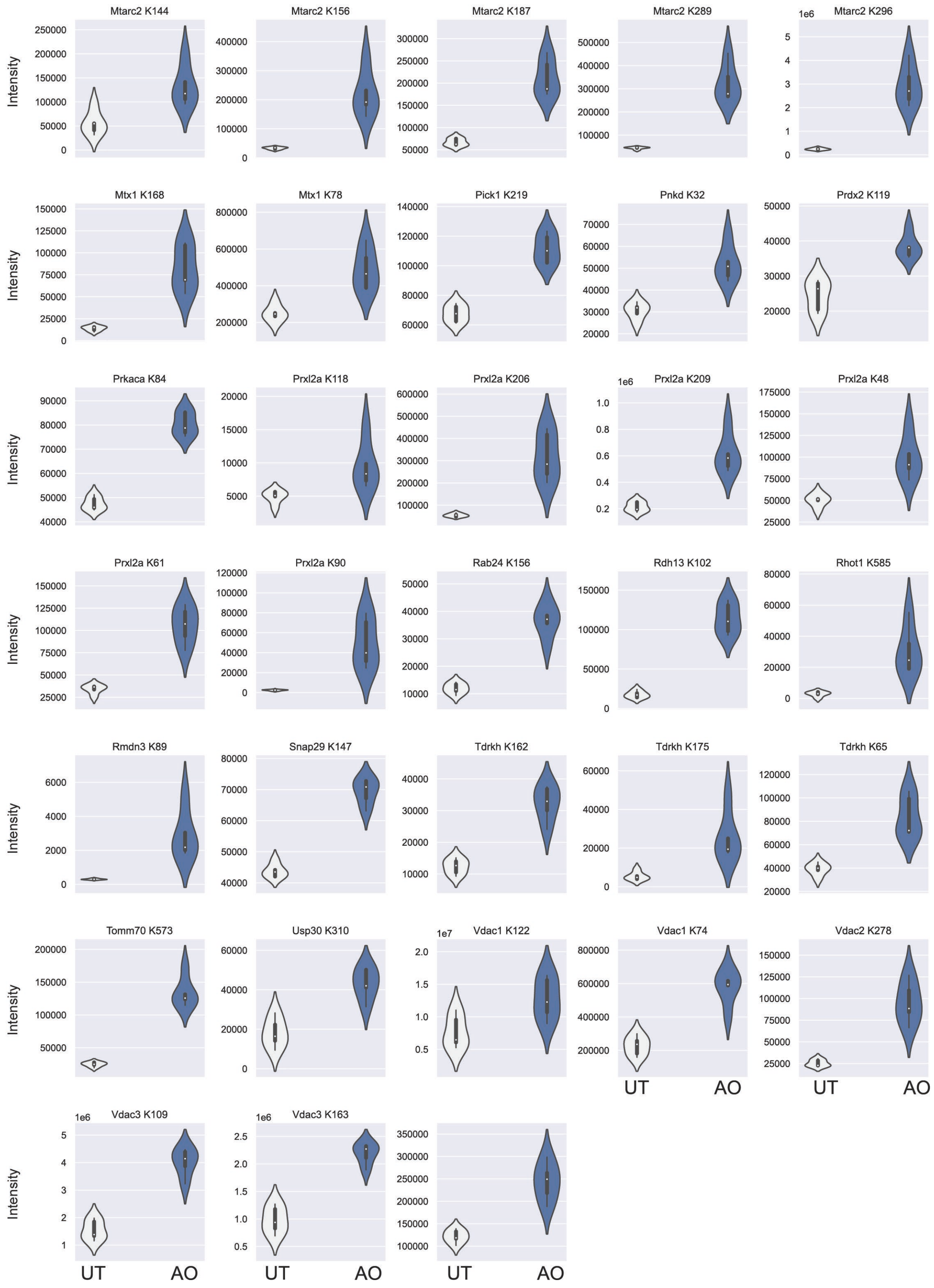

Mtarc2 K144
Mtarc2 K156
Mtarc2 K187
Mtarc2 K289
Mtarc2 K296
Mtx1 K168
Mtx1 K78
Pick1 K219
Pnkd K32
Prdx2 K119
Prkaca K84
Prxl2a K118
Prxl2a K206
Prxl2a K209
Prxl2a K48
Prxl2a K61
Prxl2a K90
Rab24 K156
Rdh13 K102
Rhot1 K585
Rmdn3 K89
Snap29 K147
Tdrkh K162
Tdrkh K175
Tdrkh K65
Tomm70 K573
Usp30 K310
Vdac1 K122
Vdac1 K74
Vdac2 K278
Vdac3 K109
Vdac3 K163
Intensity
UT
AO

SFig 11C

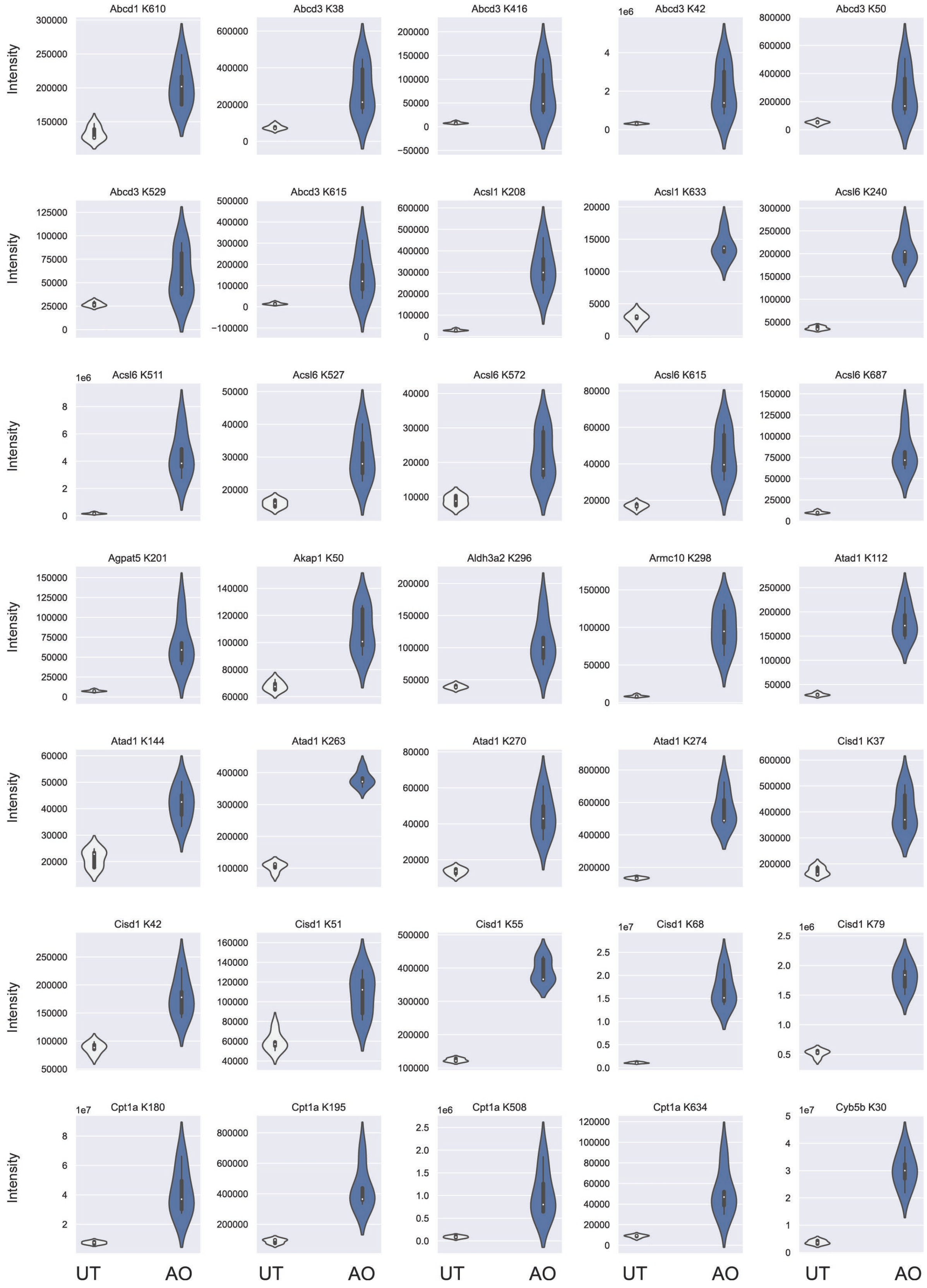
Abcd1 K610
Abcd3 K38
Abcd3 K416
Abcd3 K42
Abcd3 K50
Abcd3 K529
Abcd3 K615
Acsl1 K208
Acsl1 K633
Acsl6 K240
Acsl6 K511
Acsl6 K527
Acsl6 K572
Acsl6 K615
Acsl6 K687
Agpat5 K201
Akap1 K50
Aldh3a2 K296
Armc10 K298
Atad1 K112
Atad1 K144
Atad1 K263
Atad1 K270
Atad1 K274
Cisd1 K37
Cisd1 K42
Cisd1 K51
Cisd1 K55
Cisd1 K68
Cisd1 K79
Cpt1a K180
Cpt1a K195
Cpt1a K508
Cpt1a K634
Cyb5b K30
Intensity
UT
AO

SFig 11D

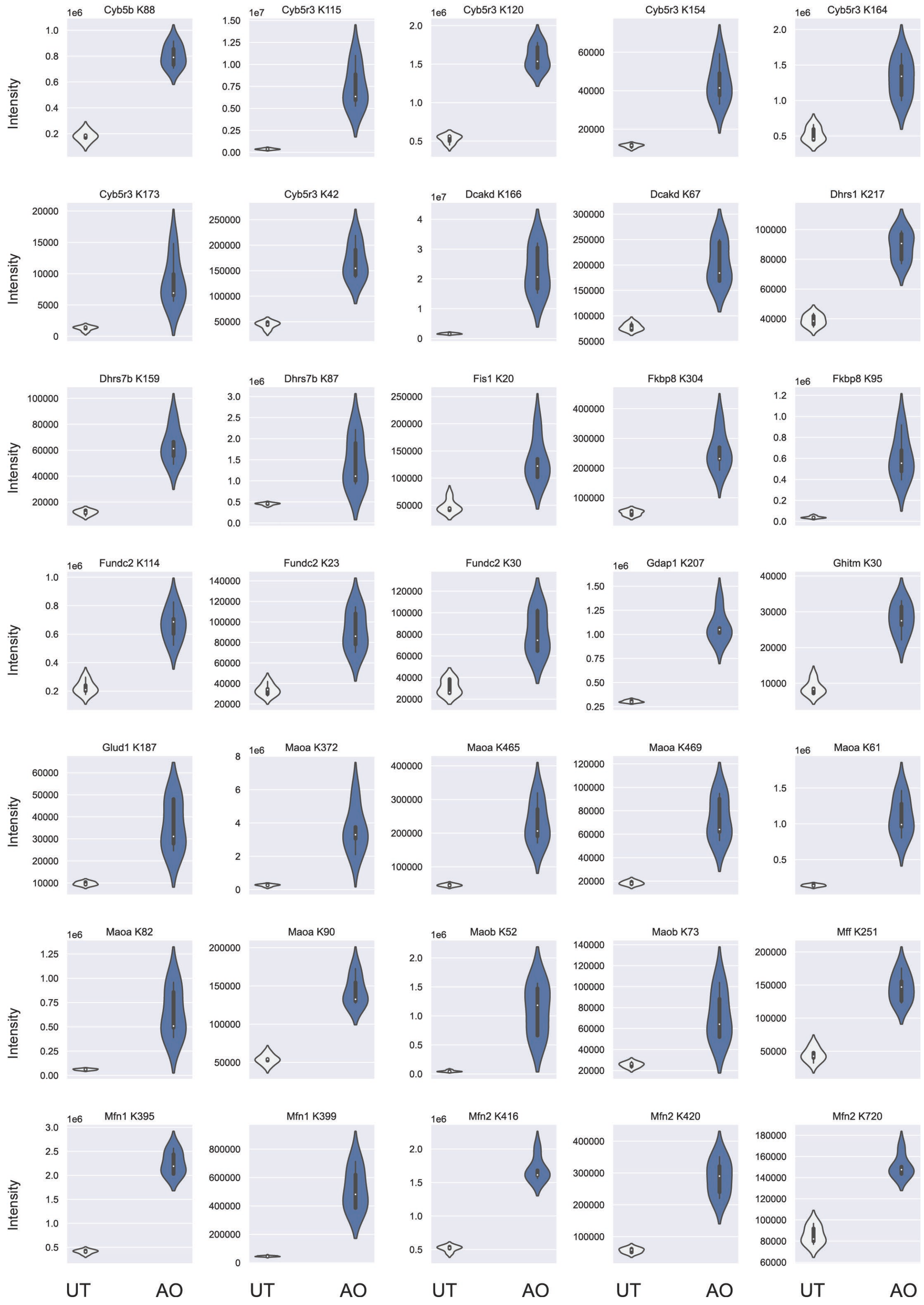
1e6 Cyb5b K88
1e7 Cyb5r3 K115
1e6 Cyb5r3 K120
Cyb5r3 K154
1e6 Cyb5r3 K164
Cyb5r3 K173
Cyb5r3 K42
1e7 Dcakd K166
Dcakd K67
Dhrs1 K217
Dhrs7b K159
1e6 Dhrs7b K87
Fis1 K20
Fkbp8 K304
1e6 Fkbp8 K95
1e6 Fundc2 K114
Fundc2 K23
Fundc2 K30
1e6 Gdap1 K207
Ghitm K30
Glud1 K187
1e6 Maoa K372
Maoa K465
Maoa K469
1e6 Maoa K61
1e6 Maoa K82
Maoa K90
1e6 Maob K52
Maob K73
Mff K251
1e6 Mfn1 K395
Mfn1 K399
1e6 Mfn2 K416
Mfn2 K420
Mfn2 K720
Intensity
UT
AO

SFig 11E

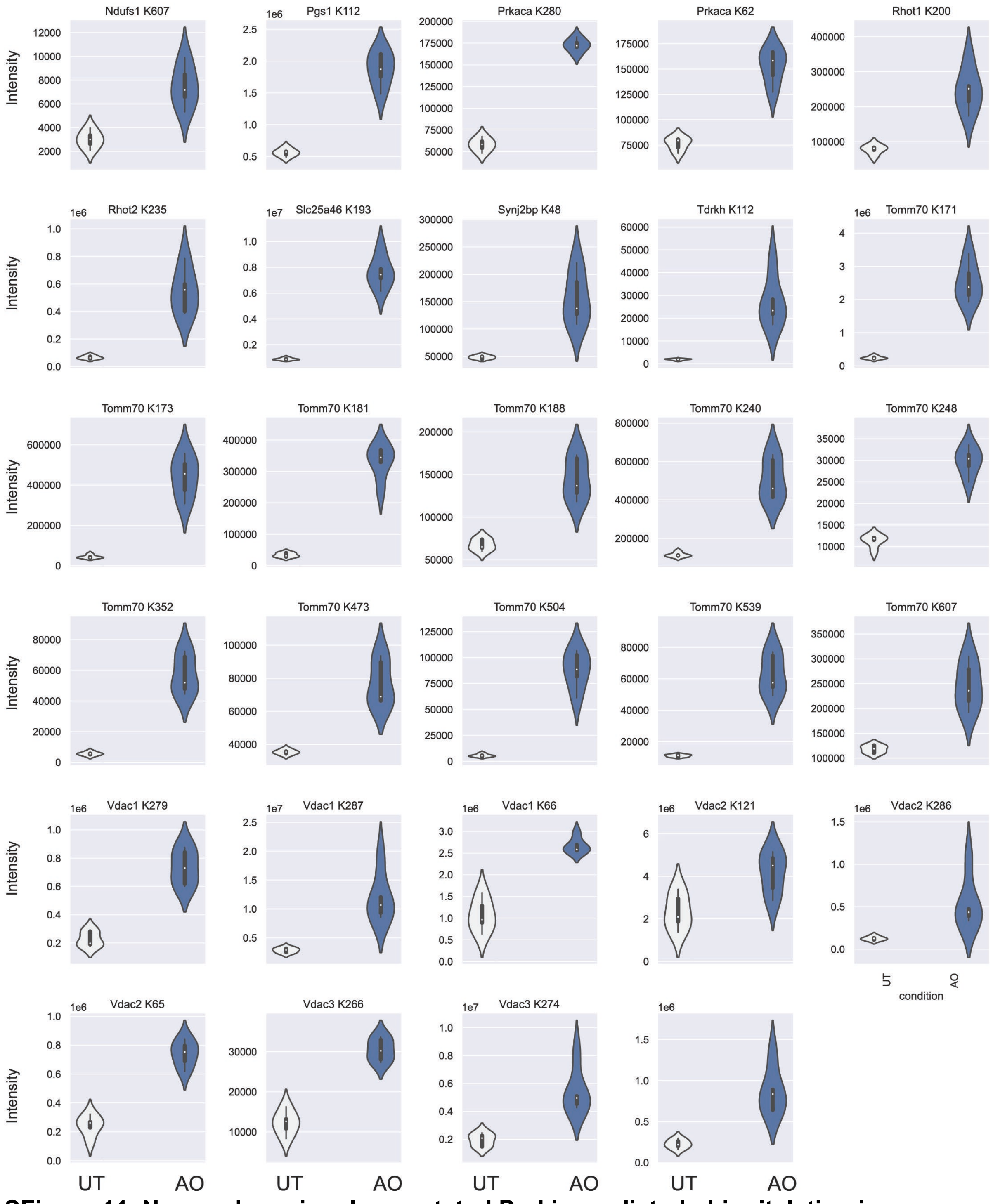

SFigure 11: **New and previously annotated Parkin mediated ubiquitylation in neurons**. Grid of violin plots of significantly changed proteins with log2 fold change greater than 1.0 derived from the experiments described in Figure 8A. (A & B) Novel sites highlighted by the CURTAIN-PTM analysis. (C to E). Previously highlighted sites {Antico, 2021 #6252}.

Supplemental dataset legends:

Dataset S1:

Tabulated dataset containing description and parameters for files input of (PPM1H) data for CURTAIN and CURTAIN-PTM. The parameter name as they appear on the web interface is in the “Parameters” column while further description of these parameters can be found in the “Description” column. The column names that have been selected as input are found in the “Column names”.

Dataset S2:

Tabulated text file containing all currently available data filter lists that can be used for batch selection within CURTAIN and CURTAIN-PTM. The title of the list is located in the “name” column. The biomolecular group/association category the data belong to is located in the “category” column. The individual protein or gene name in the list can be found in the “data” column delimited within the column by a “;”.

Dataset S3:

Tabulated dataset containing all the modification types (“Modification Type” column) and the name of the databases (“Database Name” column) the modifications.

Dataset S4:

Tabulated dataset containing the data used as input for CURTAIN and CURTAIN-PTM analysis of PPM1H-BromTAG experiment. The differential analysis for CURTAIN could be found in the “PPM1H-BromTAG_Total proteome” sheet while the data for CURTAIN-PTM could be found in the “PPM1H-BromTAG_Phosphoproteome” sheet.

Dataset S5:

Tabulated dataset containing the data used as input for CURTAIN-PTM analysis of the ubiquitinome of WT-PINK1 and PINK1-KO mouse primary cortical neurons that are treated ± Antimycin-A/Oligomycin experiment. The data for analysis of WT-PINK1 mouse primary cortical neurons that are treated ± Antimycin-A/Oligomycin (AO) could be found in the “tTest_WT-AO_UT”sheet while the data for analysis of WT-PINK1 and PINK1-KO mouse primary cortical neurons that are treated ± Antimycin-A/Oligomycin (AO)could be found in the “tTest_WT-KO_AO_UT” sheet.

Dataset S6.

Tabulated dataset containing the package and software dependencies used in the development of various components of CURTAIN and CURTAIN-PTM.

Dataset S7.

Comparison of CURTAIN and CURTAIN-PTM tools features with few open-source and commercial complimentary tools.

Source code:

Source code 1: Curtain frontend.

Source code 2: CURTAIN-PTM frontend.

Source code 3: Curtain backend.

Source code 4: Curtain JavaScript API.

Source code 5: Curtain python utility package.

Source code 6: Uniprot parser JavaScript package.

Source code 7: Python script for generating custom PTM database.
