# Supplementary material for: CURTAIN—A unique web-based tool for exploration and sharing of MS-based proteomics data
Source: Proc Natl Acad Sci U S A. 2024 Feb 7;121(7):e2312676121. doi: 10.1073/pnas.2312676121 (PMC10873628; doi:10.1073/pnas.2312676121)
Supplement: Supplementary file 9 — Code S01 (ZIP) [file pnas.2312676121.sd08.zip › Alessi-Lab-curtain-353715d/src/app/accounts/accounts/accounts.component.html]

##### Account Information

**Username:** {{accounts.curtainAPI.user.username}}  
**Curtain Link Limits:** infinite{{accounts.curtainAPI.user.curtainLinkLimit}} - Limit Exceed   
**Total Curtain Sessions:**  {{accounts.curtainAPI.user.totalCurtain}}

Search Session Description

Search


{{selectedCount}} Selected

|  | Link | Description | Public or Private | Actions Add Owner  Submit Remove Selected Set Selected Public Set Selected Private |
| --- | --- | --- | --- | --- |
|  |  |  |  |  |
| --- | --- | --- | --- | --- |
|  | {{d.link\_id}} |  | Public  Private | Details Rm |
| **Owners:**- {{o.username}} |
|
