# Supplementary material for: CURTAIN—A unique web-based tool for exploration and sharing of MS-based proteomics data
Source: Proc Natl Acad Sci U S A. 2024 Feb 7;121(7):e2312676121. doi: 10.1073/pnas.2312676121 (PMC10873628; doi:10.1073/pnas.2312676121)
Supplement: Supplementary file 9 — Code S01 (ZIP) [file pnas.2312676121.sd08.zip › Alessi-Lab-curtain-353715d/src/app/components/bar-chart/bar-chart.component.html]

Download Plot

Download Bar Chart
Download Average Bar Chart
Download Violin Plot
Download All Plots

Standard Error
Standard Deviation

Dot points within error bar chart

Select conditions


{{c}}

Condition A


{{c}}

Condition B


{{c}}

Test Type


{{d}}

Perform Test

| Conditions | Score |
| --- | --- |
|  |  |  |
| --- | --- | --- |
| {{c.conditions.join(",")}} | {{c.comparison.f | number}} | {{c.comparison.p\_value | number}} |
