# Supplementary material for: CURTAIN—A unique web-based tool for exploration and sharing of MS-based proteomics data
Source: Proc Natl Acad Sci U S A. 2024 Feb 7;121(7):e2312676121. doi: 10.1073/pnas.2312676121 (PMC10873628; doi:10.1073/pnas.2312676121)
Supplement: Supplementary file 9 — Code S01 (ZIP) [file pnas.2312676121.sd08.zip › Alessi-Lab-curtain-353715d/src/app/components/batch-search/batch-search.component.html]

### Protein Batch Search

-
Or
-

Builtin category


{{c}}

Builtin sub-category


{{c.name}}

Identifier type


Gene Names

Primary IDs

Select only significant data according to current volcano plot settings

Save

Delete

---

Enable search within fold change and significant values range

Enable search on the left side of the volcano plot

Minimum abs(log2FC) on the left side of the volcano plot

Maximum abs(log2FC) on the left side of the volcano plot

Enable search on the right side of the volcano plot

Minimum abs(log2FC) on the right of the volcano plot

Maximum abs(log2FC) on the right of the volcano plot

Minimum significant value

Max significant value

Submit
Close
