# Supplementary material for: CURTAIN—A unique web-based tool for exploration and sharing of MS-based proteomics data
Source: Proc Natl Acad Sci U S A. 2024 Feb 7;121(7):e2312676121. doi: 10.1073/pnas.2312676121 (PMC10873628; doi:10.1073/pnas.2312676121)
Supplement: Supplementary file 9 — Code S01 (ZIP) [file pnas.2312676121.sd08.zip › Alessi-Lab-curtain-353715d/src/app/components/citation/citation.component.html]

Resource Citation

The UniProt Consortium   
UniProt: the universal protein knowledgebase in 2021   
Nucleic Acids Res. 49:D1 (2021)


Samaras et al., ProteomicsDB: a multi-omics and multi-organism resource for life science research. Nucleic Acids Res. 2019 Oct 30  
Schmidt, Samaras et al., ProteomicsDB. Nucleic Acids Res. 2017 Nov 2  


Szklarczyk D\*, Gable AL\*, Nastou KC, Lyon D, Kirsch R, Pyysalo S, Doncheva NT, Legeay M, Fang T, Bork P‡, Jensen LJ‡, von Mering C‡.   
The STRING database in 2021: customizable protein–protein networks, and functional characterization of user-uploaded gene/measurement sets .  
Nucleic Acids Res. 2021 Jan 8;49(D1):D605-12.  
  
Szklarczyk D, Gable AL, Lyon D, Junge A, Wyder S, Huerta-Cepas J, Simonovic M, Doncheva NT, Morris JH, Bork P‡, Jensen LJ‡, von Mering C‡.  
STRING v11: protein-protein association networks with increased coverage, supporting functional discovery in genome-wide experimental datasets.  
Nucleic Acids Res. 2019 Jan; 47:D607-613.  
  
Szklarczyk D, Morris JH, Cook H, Kuhn M, Wyder S, Simonovic M, Santos A, Doncheva NT, Roth A, Bork P‡, Jensen LJ‡, von Mering C‡.  
The STRING database in 2017: quality-controlled protein-protein association networks, made broadly accessible.  
Nucleic Acids Res. 2017 Jan; 45:D362-68.  
  
Szklarczyk D, Franceschini A, Wyder S, Forslund K, Heller D, Huerta-Cepas J, Simonovic M, Roth A, Santos A, Tsafou KP, Kuhn M, Bork P‡, Jensen LJ‡, von Mering C‡.  
STRING v10: protein-protein interaction networks, integrated over the tree of life.  
  
Nucleic Acids Res. 2015 Jan; 43:D447-52.   
  
Franceschini A, Lin J, von Mering C, Jensen LJ‡.  
SVD-phy: improved prediction of protein functional associations through singular value decomposition of phylogenetic profiles.  
Bioinformatics. 2015 Nov; btv696.  
  
Franceschini A\*, Szklarczyk D\*, Frankild S\*, Kuhn M, Simonovic M, Roth A, Lin J, Minguez P, Bork P‡, von Mering C‡, Jensen LJ‡.  
STRING v9.1: protein-protein interaction networks, with increased coverage and integration.  
Nucleic Acids Res. 2013 Jan; 41:D808-15.  
  
Szklarczyk D\*, Franceschini A\*, Kuhn M\*, Simonovic M, Roth A, Minguez P, Doerks T, Stark M, Muller J, Bork P‡, Jensen LJ‡, von Mering C‡.  
The STRING database in 2011: functional interaction networks of proteins, globally integrated and scored.  
Nucleic Acids Res. 2011 Jan; 39:D561-8.  
  
Jensen LJ\*, Kuhn M\*, Stark M, Chaffron S, Creevey C, Muller J, Doerks T, Julien P, Roth A, Simonovic M, Bork P‡, von Mering C‡.  
STRING 8--a global view on proteins and their functional interactions in 630 organisms.  
Nucleic Acids Res. 2009 Jan; 37:D412-6.  
  
von Mering C\*, Jensen LJ\*, Kuhn M, Chaffron S, Doerks T, Krueger B, Snel B, Bork P‡.  
STRING 7--recent developments in the integration and prediction of protein interactions.  
Nucleic Acids Res. 2007 Jan; 35:D358-62.  
  
von Mering C, Jensen LJ, Snel B, Hooper SD, Krupp M, Foglierini M, Jouffre N, Huynen MA, Bork P‡.  
STRING: known and predicted protein-protein associations, integrated and transferred across organisms.  
Nucleic Acids Res. 2005 Jan; 33:D433-7.  
  
von Mering C, Huynen M, Jaeggi D, Schmidt S, Bork P‡, Snel B.  
STRING: a database of predicted functional associations between proteins.  
Nucleic Acids Res. 2003 Jan; 31:258-61.  
  
Snel B‡, Lehmann G, Bork P, Huynen MA.  
STRING: a web-server to retrieve and display the repeatedly occurring neighbourhood of a gene.  
Nucleic Acids Res. 2000 Sep 15;28(18):3442-4.  
  
\*contributed equally  
‡corresponding author  


Luck, K., Kim, DK., Lambourne, L. et al. A reference map of the human binary protein interactome. Nature 580, 402–408 (2020). https://doi.org/10.1038/s41586-020-2188-x  
  
Rolland T, Taşan M, Charloteaux B, Pevzner SJ, Zhong Q, Sahni N, Yi S, Lemmens I, Fontanillo C, Mosca R, Kamburov A, Ghiassian SD, Yang X, Ghamsari L, Balcha D, Begg BE, Braun P, Brehme M, Broly MP, Carvunis AR, Convery-Zupan D, Corominas R, Coulombe-Huntington J, Dann E, Dreze M, Dricot A, Fan C, Franzosa E, Gebreab F, Gutierrez BJ, Hardy MF, Jin M, Kang S, Kiros R, Lin GN, Luck K, MacWilliams A, Menche J, Murray RR, Palagi A, Poulin MM, Rambout X, Rasla J, Reichert P, Romero V, Ruyssinck E, Sahalie JM, Scholz A, Shah AA, Sharma A, Shen Y, Spirohn K, Tam S, Tejeda AO, Trigg SA, Twizere JC, Vega K, Walsh J, Cusick ME, Xia Y, Barabási AL, Iakoucheva LM, Aloy P, De Las Rivas J, Tavernier J, Calderwood MA, Hill DE, Hao T, Roth FP, Vidal M. A proteome-scale map of the human interactome network. Cell. 2014 Nov 20;159(5):1212-1226. doi: 10.1016/j.cell.2014.10.050. PMID: 25416956; PMCID: PMC4266588.  
  
Yang X, Coulombe-Huntington J, Kang S, Sheynkman GM, Hao T, Richardson A, Sun S, Yang F, Shen YA, Murray RR, Spirohn K, Begg BE, Duran-Frigola M, MacWilliams A, Pevzner SJ, Zhong Q, Trigg SA, Tam S, Ghamsari L, Sahni N, Yi S, Rodriguez MD, Balcha D, Tan G, Costanzo M, Andrews B, Boone C, Zhou XJ, Salehi-Ashtiani K, Charloteaux B, Chen AA, Calderwood MA, Aloy P, Roth FP, Hill DE, Iakoucheva LM, Xia Y, Vidal M. Widespread Expansion of Protein Interaction Capabilities by Alternative Splicing. Cell. 2016 Feb 11;164(4):805-17. doi: 10.1016/j.cell.2016.01.029. PMID: 26871637; PMCID: PMC4882190.  
  
Yu H, Tardivo L, Tam S, Weiner E, Gebreab F, Fan C, Svrzikapa N, Hirozane-Kishikawa T, Rietman E, Yang X, Sahalie J, Salehi-Ashtiani K, Hao T, Cusick ME, Hill DE, Roth FP, Braun P, Vidal M. Next-generation sequencing to generate interactome datasets. Nat Methods. 2011 Jun;8(6):478-80. doi: 10.1038/nmeth.1597. Epub 2011 Apr 24. PMID: 21516116; PMCID: PMC3188388.  
  
Venkatesan K, Rual JF, Vazquez A, Stelzl U, Lemmens I, Hirozane-Kishikawa T, Hao T, Zenkner M, Xin X, Goh KI, Yildirim MA, Simonis N, Heinzmann K, Gebreab F, Sahalie JM, Cevik S, Simon C, de Smet AS, Dann E, Smolyar A, Vinayagam A, Yu H, Szeto D, Borick H, Dricot A, Klitgord N, Murray RR, Lin C, Lalowski M, Timm J, Rau K, Boone C, Braun P, Cusick ME, Roth FP, Hill DE, Tavernier J, Wanker EE, Barabási AL, Vidal M. An empirical framework for binary interactome mapping. Nat Methods. 2009 Jan;6(1):83-90. doi: 10.1038/nmeth.1280. Epub 2008 Dec 7. PMID: 19060904; PMCID: PMC2872561.  
  
Rual JF, Venkatesan K, Hao T, Hirozane-Kishikawa T, Dricot A, Li N, Berriz GF, Gibbons FD, Dreze M, Ayivi-Guedehoussou N, Klitgord N, Simon C, Boxem M, Milstein S, Rosenberg J, Goldberg DS, Zhang LV, Wong SL, Franklin G, Li S, Albala JS, Lim J, Fraughton C, Llamosas E, Cevik S, Bex C, Lamesch P, Sikorski RS, Vandenhaute J, Zoghbi HY, Smolyar A, Bosak S, Sequerra R, Doucette-Stamm L, Cusick ME, Hill DE, Roth FP, Vidal M. Towards a proteome-scale map of the human protein-protein interaction network. Nature. 2005 Oct 20;437(7062):1173-8. doi: 10.1038/nature04209. Epub 2005 Sep 28. PMID: 16189514.  
  


Jumper, J et al. Highly accurate protein structure prediction with AlphaFold (Nature 2021).


David Sehnal, Sebastian Bittrich, Mandar Deshpande, Radka Svobodová, Karel Berka, Václav Bazgier, Sameer Velankar, Stephen K Burley, Jaroslav Koča, Alexander S Rose: Mol\* Viewer: modern web app for 3D visualization and analysis of large biomolecular structures, Nucleic Acids Research, 2021; 10.1093/nar/gkab31.


AlphaFold Protein Structure Database: massively expanding the structural coverage of protein-sequence space with high-accuracy models.  
Varadi M, Anyango S, Deshpande M, Nair S, Natassia C, Yordanova G, Yuan D, Stroe O, Wood G, Laydon A, Žídek A, Green T, Tunyasuvunakool K, Petersen S, Jumper J, Clancy E, Green R, Vora A, Lutfi M, Figurnov M, Cowie A, Hobbs N, Kohli P, Kleywegt G, Birney E, Hassabis D, Velankar S.  
Nucleic acids research (2021)  
DOI: 10.1093/nar/gkab1061  
  
PDBe-KB: collaboratively defining the biological context of structural data.  
PDBe-KB consortium .  
Nucleic acids research (2021)  
DOI: 10.1093/nar/gkab988  
  
PDBeCIF: an open-source mmCIF/CIF parsing and processing package.  
van Ginkel G, Pravda L, Dana JM, Varadi M, Keller P, Anyango S, Velankar S.  
BMC bioinformatics Volume 22 (2021) p.383  
DOI: 10.1186/s12859-021-04271-9
