# Supplementary material for: CURTAIN—A unique web-based tool for exploration and sharing of MS-based proteomics data
Source: Proc Natl Acad Sci U S A. 2024 Feb 7;121(7):e2312676121. doi: 10.1073/pnas.2312676121 (PMC10873628; doi:10.1073/pnas.2312676121)
Supplement: Supplementary file 9 — Code S01 (ZIP) [file pnas.2312676121.sd08.zip › Alessi-Lab-curtain-353715d/src/app/components/collaborate-modal/collaborate-modal.component.html]

##### Collaborate settings

Collaborate is a feature that allows other users to jointly explore the session together. Those using the same collaborative link below will be able to interact with each other through the chat box in the button left.

Session ID

Display name

Collaborative session link: {{collaborateLink}}

Save
Cancel
