# Supplementary material for: CURTAIN—A unique web-based tool for exploration and sharing of MS-based proteomics data
Source: Proc Natl Acad Sci U S A. 2024 Feb 7;121(7):e2312676121. doi: 10.1073/pnas.2312676121 (PMC10873628; doi:10.1073/pnas.2312676121)
Supplement: Supplementary file 9 — Code S01 (ZIP) [file pnas.2312676121.sd08.zip › Alessi-Lab-curtain-353715d/src/app/components/default-color-palette/default-color-palette.component.html]

##### Default Color Palette Customization

These are the default colors that will be used for colorable components of plots that do not have a fixed color yet. These colors will be used in the order they are listed and would cycle to the begin of the list once it has reached the last color. You can customize the default color palette by selecting a built-in palette or by creating a custom palette.

| Current Palette |
| --- |
|  |

Customize Palette

| Select a built-in palette | {{o}} |
| --- | --- |
|  |  |

Customize Palette

| Custom Palette |  |
| --- | --- |
|  | - |
| + |

Clear Palette

Parse Custom Palette Config

Copy Current Palette

Reset Volcano Color

Reset Bar Chart Color

Update Color Palette

Close
