# Supplementary material for: CURTAIN—A unique web-based tool for exploration and sharing of MS-based proteomics data
Source: Proc Natl Acad Sci U S A. 2024 Feb 7;121(7):e2312676121. doi: 10.1073/pnas.2312676121 (PMC10873628; doi:10.1073/pnas.2312676121)
Supplement: Supplementary file 9 — Code S01 (ZIP) [file pnas.2312676121.sd08.zip › Alessi-Lab-curtain-353715d/src/app/components/draggable-element/draggable-element.component.html]

{{label.slice(0,15)}}

{{label}}
