# Supplementary material for: CURTAIN—A unique web-based tool for exploration and sharing of MS-based proteomics data
Source: Proc Natl Acad Sci U S A. 2024 Feb 7;121(7):e2312676121. doi: 10.1073/pnas.2312676121 (PMC10873628; doi:10.1073/pnas.2312676121)
Supplement: Supplementary file 9 — Code S01 (ZIP) [file pnas.2312676121.sd08.zip › Alessi-Lab-curtain-353715d/src/app/components/enrichr-modal/enrichr-modal.component.html]

##### Enrichr

Study gene list

{{s}}

Background list

All genes
{{s}}

Geneset library

{{l}}

Chen EY, Tan CM, Kou Y, Duan Q, Wang Z, Meirelles GV, Clark NR, Ma'ayan A.
Enrichr: interactive and collaborative HTML5 gene list enrichment analysis tool. BMC Bioinformatics. 2013; 128(14).

Kuleshov MV, Jones MR, Rouillard AD, Fernandez NF, Duan Q, Wang Z, Koplev S, Jenkins SL, Jagodnik KM, Lachmann A, McDermott MG, Monteiro CD, Gundersen GW, Ma'ayan A.
Enrichr: a comprehensive gene set enrichment analysis web server 2016 update. Nucleic Acids Research. 2016; gkw377 .

Xie Z, Bailey A, Kuleshov MV, Clarke DJB., Evangelista JE, Jenkins SL, Lachmann A, Wojciechowicz ML, Kropiwnicki E, Jagodnik KM, Jeon M, & Ma’ayan A.
Gene set knowledge discovery with Enrichr. Current Protocols, 1, e90. 2021. doi: 10.1002/cpz1.90

Submit
Cancel
