# Supplementary material for: CURTAIN—A unique web-based tool for exploration and sharing of MS-based proteomics data
Source: Proc Natl Acad Sci U S A. 2024 Feb 7;121(7):e2312676121. doi: 10.1073/pnas.2312676121 (PMC10873628; doi:10.1073/pnas.2312676121)
Supplement: Supplementary file 9 — Code S01 (ZIP) [file pnas.2312676121.sd08.zip › Alessi-Lab-curtain-353715d/src/app/components/file-form/file-form.component.html]

## Data Parameters Input

Example session

##### Differential Analysis File

Select Primary IDs


{{c}}
Unique and should contain UniProt Accession ID for Curtain to obtain extra metadata.

Select Gene Names


{{c}}

Select Fold Change


{{c}}

Perform log2 Transformation

Reverse fold change value

Select Significant


{{c}}

Perform -log10 Transformation

Comparison


{{c}}

Select Comparison


{{c}}

##### Raw File

Select Primary IDs


{{c}}
Unique and should be the same as differential analysis primary ID column

Select Samples


{{c}}
Sample column names in format "condition"."replicate" for automated experimental condition parsing

**Session Description**

Submit

Fetch Uniprot Data Using Primary Ids

{{progressBar.text}}
