# Supplementary material for: CURTAIN—A unique web-based tool for exploration and sharing of MS-based proteomics data
Source: Proc Natl Acad Sci U S A. 2024 Feb 7;121(7):e2312676121. doi: 10.1073/pnas.2312676121 (PMC10873628; doi:10.1073/pnas.2312676121)
Supplement: Supplementary file 9 — Code S01 (ZIP) [file pnas.2312676121.sd08.zip › Alessi-Lab-curtain-353715d/src/app/components/home/home.component.html]

**Curtain**


{{uniqueLink}}

QR Code

{{progressEvent.text}}

New Curtain version available

State

Create local state
State Management

Plot

Correlation Matrix

Profile Plot ({{settings.settings.selectedComparison.length}})

Get Selected Protein List

Sample Order & Visibility Settings

Customize Color Palette
Fold Change Distribution

Session
private
shareable

Project Annotation

Save Session
Clear Selections
Download Differential Analysis File
Download Raw File
Enrichr


Data Selection Management

Sample & Condition Assignment

Compare Sessions

Account Login

Session Settings

Collaborate


Account

Logout
 Video Tutorial
 *Support Google Group*


**GDPR Statement**  

This website neither uses cookies nor tracks/logs ips of its users. The only personal identification data that we store is ORCID id so that the user can track the session they have submitted.


## Rank Abundance Plot

- Resource Citation
