# Supplementary material for: CURTAIN—A unique web-based tool for exploration and sharing of MS-based proteomics data
Source: Proc Natl Acad Sci U S A. 2024 Feb 7;121(7):e2312676121. doi: 10.1073/pnas.2312676121 (PMC10873628; doi:10.1073/pnas.2312676121)
Supplement: Supplementary file 9 — Code S01 (ZIP) [file pnas.2312676121.sd08.zip › Alessi-Lab-curtain-353715d/src/app/components/interactome-atlas/interactome-atlas.component.html]

Download PNG

No data found

| # | Name | Author | Year | Description | Status | Reference |
| --- | --- | --- | --- | --- | --- | --- |
| {{ i + 1 }} | {{s["name"]}} | {{s["dataset\_author"]}} | {{s["year"]}} | {{s["description"]}} | {{s["interaction\_status"]}} | {{s["dataset\_reference"]}} |

Enable Filter

Score cutoff:

| Nodes color | Description |
| --- | --- |
|  | Increased abundance |
|  | Decreased abundance |
|  | Protein found in data set but was not changed according to volcano plot settings |
|  | Protein not found in data set |

| Edges color | Description |
| --- | --- |
|  | HI-Union |
|  | Literature |
|  | HI-Union and Literature |

Update
