# Supplementary material for: CURTAIN—A unique web-based tool for exploration and sharing of MS-based proteomics data
Source: Proc Natl Acad Sci U S A. 2024 Feb 7;121(7):e2312676121. doi: 10.1073/pnas.2312676121 (PMC10873628; doi:10.1073/pnas.2312676121)
Supplement: Supplementary file 9 — Code S01 (ZIP) [file pnas.2312676121.sd08.zip › Alessi-Lab-curtain-353715d/src/app/components/local-session-state-modal/local-session-state-modal.component.html]

##### Local session state management

The local states in this section include settings like color, searched primary ids, annotation text without the imported data. They can be loaded independently of the session they are associated with. These states are stored within the browser local storage and can be retrieve and load indepedently as files.

Session: {{s.currentID}}   
Created: {{s.date | date:'short'}}

Download

Load

Delete

Load from state file
Close
