# Supplementary material for: CURTAIN—A unique web-based tool for exploration and sharing of MS-based proteomics data
Source: Proc Natl Acad Sci U S A. 2024 Feb 7;121(7):e2312676121. doi: 10.1073/pnas.2312676121 (PMC10873628; doi:10.1073/pnas.2312676121)
Supplement: Supplementary file 9 — Code S01 (ZIP) [file pnas.2312676121.sd08.zip › Alessi-Lab-curtain-353715d/src/app/components/pdb-viewer/pdb-viewer.component.html]

#### AlphaFold Predicted Structure

Gene name: {{geneName}}   
Entry ID: {{entryID}}   
PDB File: Download  
Model Created Date: {{modelCreatedDate}}  
Version: {{version}}  
Original Source: Link   
EBI AlphaFold Predicted Structure Database

| Color | Description |
| --- | --- |
|  | Very high (pLDDT > 90) |
|  | Confident (90 > pLDDT > 70) |
|  | Low (70 > pLDDT > 50) |
|  | Very low (pLDDT < 50) |

Close
