# Supplementary material for: CURTAIN—A unique web-based tool for exploration and sharing of MS-based proteomics data
Source: Proc Natl Acad Sci U S A. 2024 Feb 7;121(7):e2312676121. doi: 10.1073/pnas.2312676121 (PMC10873628; doi:10.1073/pnas.2312676121)
Supplement: Supplementary file 9 — Code S01 (ZIP) [file pnas.2312676121.sd08.zip › Alessi-Lab-curtain-353715d/src/app/components/pride/pride.component.html]

{{settings.settings.project.title}}

**Project Description**   


**Organism**   

- {{o.name}}

**Organism Parts**  

- {{o.name}}
**Sample Processing Protocol**   

**Data Processing Protocol**   


**Identified PTMs**   

- {{s.name}}

**Sample Annotations**

| Sample ID | Sample Description |
| --- | --- |
| {{a}} | {{settings.settings.project.sampleAnnotations[a]}} |

Additional Information

- **{{a.cvLabel}}:** {{a.name}}
- **PRIDE Dataset FTP URL:**  FTP Link
- **PRIDE File URL:**  File Link
- **PRIDE Project Link:**  Project Link

Affiliations

- {{a.name}}

Print Project Description
