# Supplementary material for: CURTAIN—A unique web-based tool for exploration and sharing of MS-based proteomics data
Source: Proc Natl Acad Sci U S A. 2024 Feb 7;121(7):e2312676121. doi: 10.1073/pnas.2312676121 (PMC10873628; doi:10.1073/pnas.2312676121)
Supplement: Supplementary file 10 — Code S02 (ZIP) [file pnas.2312676121.sd09.zip › Alessi-Lab-curtainPTM-4e27155/docs/index.html]

CurtainPTM
