# Supplementary material for: CURTAIN—A unique web-based tool for exploration and sharing of MS-based proteomics data
Source: Proc Natl Acad Sci U S A. 2024 Feb 7;121(7):e2312676121. doi: 10.1073/pnas.2312676121 (PMC10873628; doi:10.1073/pnas.2312676121)
Supplement: Supplementary file 10 — Code S02 (ZIP) [file pnas.2312676121.sd09.zip › Alessi-Lab-curtainPTM-4e27155/src/app/components/data-block/data-block.component.html]

### {{title}}

**Variant: {{sourceMap["Experimental Data"]}}**

**UniProt Accession:** {{uni["Entry"]}}

**Protein names:** {{uni["Protein names"]}}

**Subcellular location:**- {{subCell}}


PTM identified:

- {{u.residue}}{{u.position}} {{u.id}}

Go to top

- Protein Information
- Raw Data Distribution


  Error: {{error}}
- PTM Position Viewer
