# Supplementary material for: CURTAIN—A unique web-based tool for exploration and sharing of MS-based proteomics data
Source: Proc Natl Acad Sci U S A. 2024 Feb 7;121(7):e2312676121. doi: 10.1073/pnas.2312676121 (PMC10873628; doi:10.1073/pnas.2312676121)
Supplement: Supplementary file 10 — Code S02 (ZIP) [file pnas.2312676121.sd09.zip › Alessi-Lab-curtainPTM-4e27155/src/app/components/data-viewer/data-viewer.component.html]

---


Gene Names

Primary IDs

Involvement in Diseases (UniProt)


---

---


---

##### No data found

Please try a different search term.
