# Supplementary material for: CURTAIN—A unique web-based tool for exploration and sharing of MS-based proteomics data
Source: Proc Natl Acad Sci U S A. 2024 Feb 7;121(7):e2312676121. doi: 10.1073/pnas.2312676121 (PMC10873628; doi:10.1073/pnas.2312676121)
Supplement: Supplementary file 10 — Code S02 (ZIP) [file pnas.2312676121.sd09.zip › Alessi-Lab-curtainPTM-4e27155/src/app/components/kinase-info/kinase-info.component.html]

### Kinase {{\_uni["Gene Names"]}}

**UniProt Acc:** {{\_uni["Entry"]}}  
**Gene name:** {{\_uni["Gene Names"]}}  
**Protein names:** {{otherUni["proteinDescription"]["recommendedName"]["fullName"]["value"]}}

**Functional description:**

- {{f.replace('FUNCTION: ', '').replace('; ', '')}}

Close
