# Supplementary material for: CURTAIN—A unique web-based tool for exploration and sharing of MS-based proteomics data
Source: Proc Natl Acad Sci U S A. 2024 Feb 7;121(7):e2312676121. doi: 10.1073/pnas.2312676121 (PMC10873628; doi:10.1073/pnas.2312676121)
Supplement: Supplementary file 10 — Code S02 (ZIP) [file pnas.2312676121.sd09.zip › Alessi-Lab-curtainPTM-4e27155/src/app/components/kinase-library-modal/kinase-library-modal.component.html]

Kinase Library

**Kinase Library Web-link:** {{sequenceWindow}}

| Kinase | Percentile | Rank |
| --- | --- | --- |
| {{i.kinase}} | {{i.percentile}} | {{i.rank}} |

No data was found in Kinase Library database. You can use the link above instead or use the database which utilize Kinase Library website API for prediction of kinase.

| Kinase | Score | Rank |
| --- | --- | --- |
| {{i.motif.geneName}} | {{i.score}} | {{i.scoreRank}} |

Close
