# Supplementary material for: CURTAIN—A unique web-based tool for exploration and sharing of MS-based proteomics data
Source: Proc Natl Acad Sci U S A. 2024 Feb 7;121(7):e2312676121. doi: 10.1073/pnas.2312676121 (PMC10873628; doi:10.1073/pnas.2312676121)
Supplement: Supplementary file 10 — Code S02 (ZIP) [file pnas.2312676121.sd09.zip › Alessi-Lab-curtainPTM-4e27155/src/app/components/navbar/navbar.component.html]

**Curtain****PTM**

{{uniqueLink}}

QR Code

New CurtainPTM version available

{{progressEvent.text}}

State

Create local state
State Management

Plot

Profile Plot ({{settings.settings.selectedComparison.length}})

Sample Order & Visibility Settings

Customize Color Palette

Session
private
shareable

Save Session
Clear Selections
Download Differential Analysis File
Download Raw File
Project Annotation

Get Selected Primary IDs List
Session QR Code

Data Selection Management

Sample & Condition Assignment

Account Login

Session Settings

Custom PTM Import Management


Account

Logout
Collaborate
 Video Tutorial
 *Support Google Group*
