# Supplementary material for: CURTAIN—A unique web-based tool for exploration and sharing of MS-based proteomics data
Source: Proc Natl Acad Sci U S A. 2024 Feb 7;121(7):e2312676121. doi: 10.1073/pnas.2312676121 (PMC10873628; doi:10.1073/pnas.2312676121)
Supplement: Supplementary file 10 — Code S02 (ZIP) [file pnas.2312676121.sd09.zip › Alessi-Lab-curtainPTM-4e27155/src/app/components/network-interactions/network-interactions.component.html]

##### Network Parameters

##### StringDB

StringDB Network Type


Functional

Physical

Required Combined Score (Max = 1)

Required T-Score Score (Max = 1)


Required A-Score Score (Max = 1)

Required D-Score Score (Max = 1)

Required E-Score Score (Max = 1)


Required F-Score Score (Max = 1)

Required N-Score Score (Max = 1)

Required P-Score Score (Max = 1)

Interactome Required Score (Max = 1)

Redraw

Save current network configuration
Download PNG

| Color | Description |
| --- | --- |
|  | Fold change > {{settings.settings.log2FCCutoff}} |
|  | Fold change < {{-settings.settings.log2FCCutoff}} |
|  | {{-settings.settings.log2FCCutoff}} < Fold change < {{settings.settings.log2FCCutoff}} |
|  | pvalue <= {{settings.settings.pCutoff}} |
|  | pvalue > {{settings.settings.pCutoff}} |
|  | StringDB interaction |
|  | Interactome Atlas interaction |

| Properties | Values |
| --- | --- |
|  |  |
| --- | --- |
| Node A | {{edgeDataViewer[edgeDataSource]["preferredName\_A"]}} |
| Node B | {{edgeDataViewer[edgeDataSource]["preferredName\_B"]}} |
| A-score | {{edgeDataViewer[edgeDataSource]["ascore"]}} |
| D-score | {{edgeDataViewer[edgeDataSource]["dscore"]}} |
| E-score | {{edgeDataViewer[edgeDataSource]["escore"]}} |
| F-score | {{edgeDataViewer[edgeDataSource]["fscore"]}} |
| N-score | {{edgeDataViewer[edgeDataSource]["nscore"]}} |
| P-score | {{edgeDataViewer[edgeDataSource]["pscore"]}} |
| T-score | {{edgeDataViewer[edgeDataSource]["tscore"]}} |
| Score | {{edgeDataViewer[edgeDataSource]["score"]}} |

|  |  |
| --- | --- |
| Node A | {{edgeDataViewer[edgeDataSource]["interactor\_A"]["protein\_gene\_name"]}} |
| Node B | {{edgeDataViewer[edgeDataSource]["interactor\_B"]["protein\_gene\_name"]}} |
| Score | {{edgeDataViewer[edgeDataSource]["score"]}} |
