# Supplementary material for: CURTAIN—A unique web-based tool for exploration and sharing of MS-based proteomics data
Source: Proc Natl Acad Sci U S A. 2024 Feb 7;121(7):e2312676121. doi: 10.1073/pnas.2312676121 (PMC10873628; doi:10.1073/pnas.2312676121)
Supplement: Supplementary file 9 — Code S01 (ZIP) [file pnas.2312676121.sd08.zip › Alessi-Lab-curtain-353715d/src/app/components/qrcode-modal/qrcode-modal.component.html]

QR Code

Scan the QR code to open the link on your mobile device.

Mobile devices with low total system RAM or low amount of allocatable RAM
for web process (iOS) might have problem with loading a large dataset in Curtain

Close
