# Supplementary material for: CURTAIN—A unique web-based tool for exploration and sharing of MS-based proteomics data
Source: Proc Natl Acad Sci U S A. 2024 Feb 7;121(7):e2312676121. doi: 10.1073/pnas.2312676121 (PMC10873628; doi:10.1073/pnas.2312676121)
Supplement: Supplementary file 9 — Code S01 (ZIP) [file pnas.2312676121.sd08.zip › Alessi-Lab-curtain-353715d/src/app/components/rank-plot-text-annotation/rank-plot-text-annotation.component.html]

Rank Plot Text Annotation Editor

##### {{f.value["annotationID"]}}

Show Annotation

Show Arrow

Arrow Head Type

Arrow Size

Arrow Width

X axis offset

Y axis offset

Font Size

Font Color

Annotation Text


---

Submit
Close
