# Supplementary material for: CURTAIN—A unique web-based tool for exploration and sharing of MS-based proteomics data
Source: Proc Natl Acad Sci U S A. 2024 Feb 7;121(7):e2312676121. doi: 10.1073/pnas.2312676121 (PMC10873628; doi:10.1073/pnas.2312676121)
Supplement: Supplementary file 9 — Code S01 (ZIP) [file pnas.2312676121.sd08.zip › Alessi-Lab-curtain-353715d/src/app/components/raw-data-block/raw-data-block.component.html]

### {{title}}

**Found in searches:**

**Primary ID:** {{primaryID}}

**UniProt Accession:** {{uni["Entry"]}}

**Protein names:** {{uni["Protein names"]}}

**Subcellular location:**- {{subCell}}

Enrichr runs:

**{{e}}**   


{{enrichrData[e][t].rank}} {{t}}


Include in profile comparison

Annotate on volcano plot

AlphaFold Structure

- Protein Information
- Raw Data Distribution
- Proteomics DB
- String DB
- Interactome Atlas

Go to top

---
