# Supplementary material for: CURTAIN—A unique web-based tool for exploration and sharing of MS-based proteomics data
Source: Proc Natl Acad Sci U S A. 2024 Feb 7;121(7):e2312676121. doi: 10.1073/pnas.2312676121 (PMC10873628; doi:10.1073/pnas.2312676121)
Supplement: Supplementary file 9 — Code S01 (ZIP) [file pnas.2312676121.sd08.zip › Alessi-Lab-curtain-353715d/src/app/components/raw-data-viewer/raw-data-viewer.component.html]

---


Gene Names

Primary IDs

Involvement in Diseases (UniProt)

Sort settings

Gene Names

Primary IDs

Fold Change

P-Value

Enrichr

Ascending

Descending

{{run}}

Sort


---

---

##### No data found

Please try a different search term.
