# Supplementary material for: CURTAIN—A unique web-based tool for exploration and sharing of MS-based proteomics data
Source: Proc Natl Acad Sci U S A. 2024 Feb 7;121(7):e2312676121. doi: 10.1073/pnas.2312676121 (PMC10873628; doi:10.1073/pnas.2312676121)
Supplement: Supplementary file 9 — Code S01 (ZIP) [file pnas.2312676121.sd08.zip › Alessi-Lab-curtain-353715d/src/app/components/sample-annotation/sample-annotation.component.html]

### Project Annotation

**PRIDE Accession ID**

Parse PRIDE Data

**Project Title**

**Project Description**

**Organisms**

+

**Organism Parts**

+

**Diseases**

+

**Cell types**

+

**Sample Processing Protocol**

**Data Processing Protocol**

**Instruments**

+

**Qualification Methods**

+

**Experiment Types**

+

**Detected PTM**

+

**Project Tags**

+

**Software**

+

**Affiliations**

+

**Authors**

+

| Sample | Annotation |
| --- | --- |
| {{s}} |  |

Submit
Close
