# Supplementary material for: CURTAIN—A unique web-based tool for exploration and sharing of MS-based proteomics data
Source: Proc Natl Acad Sci U S A. 2024 Feb 7;121(7):e2312676121. doi: 10.1073/pnas.2312676121 (PMC10873628; doi:10.1073/pnas.2312676121)
Supplement: Supplementary file 9 — Code S01 (ZIP) [file pnas.2312676121.sd08.zip › Alessi-Lab-curtain-353715d/src/app/components/sample-order-and-hide/sample-order-and-hide.component.html]

Sample Settings

**Sample Order**

Check All

Uncheck All

**{{c}}**

↑

↓

| Sample name | Move | Visibility |
| --- | --- | --- |
|  |  |  |
| --- | --- | --- |
| {{s}} | ↑  ↓ |  |

Submit

Close
