# Supplementary material for: CURTAIN—A unique web-based tool for exploration and sharing of MS-based proteomics data
Source: Proc Natl Acad Sci U S A. 2024 Feb 7;121(7):e2312676121. doi: 10.1073/pnas.2312676121 (PMC10873628; doi:10.1073/pnas.2312676121)
Supplement: Supplementary file 9 — Code S01 (ZIP) [file pnas.2312676121.sd08.zip › Alessi-Lab-curtain-353715d/src/app/components/session-comparison-result-viewer-modal/session-comparison-result-viewer-modal.component.html]

##### Session Comparison Viewer

{{s}}


Filter Term

Term Category

Primary ID
Gene Name

Source Comparison

{{c}}

Target Comparison

{{c}}

Filter
Export
Reset Filter

| Primary ID | Gene Names | Source Comparison | Source Fold Change | Source Significant | Target Fold Change | Target Significant | Target Comparison | Target Primary ID |
| --- | --- | --- | --- | --- | --- | --- | --- | --- |
| {{row.primaryID}} | {{row.geneName}} | {{row.comparison}} | {{row.currentFC\|number: '0.0-4'}} | {{row.currentPValue\|number: '0.0-4'}} | {{row.targetFC\|number: '0.0-4'}} | {{row.targetPValue\|number: '0.0-4'}} | {{row.targetComparison}} | {{row.targetPrimaryID}} |

Close
