# Supplementary material for: CURTAIN—A unique web-based tool for exploration and sharing of MS-based proteomics data
Source: Proc Natl Acad Sci U S A. 2024 Feb 7;121(7):e2312676121. doi: 10.1073/pnas.2312676121 (PMC10873628; doi:10.1073/pnas.2312676121)
Supplement: Supplementary file 9 — Code S01 (ZIP) [file pnas.2312676121.sd08.zip › Alessi-Lab-curtain-353715d/src/app/components/side-float-control/side-float-control.component.html]

State updated   

Click to load save state   

{{m.message.message}}   

{{m.message.title}}   

{{m.message.title}}   

{{m.message.message}}  


Available States:- {{s}}

{{m.message.message}}  
{{senderMap[m.senderID]}} - {{m.message.timestamp|date:'short'}}
