# Supplementary material for: CURTAIN—A unique web-based tool for exploration and sharing of MS-based proteomics data
Source: Proc Natl Acad Sci U S A. 2024 Feb 7;121(7):e2312676121. doi: 10.1073/pnas.2312676121 (PMC10873628; doi:10.1073/pnas.2312676121)
Supplement: Supplementary file 9 — Code S01 (ZIP) [file pnas.2312676121.sd08.zip › Alessi-Lab-curtain-353715d/src/app/components/string-db/string-db.component.html]

Download as SVG

| Node Label Color | Description |
| --- | --- |
|  | Increase |
|  | Decrease |
|  | In dataset |
|  | Not in dataset |

Minimum Required Score (Max = 1000)

Network Type


Functional

Physical

Network Flavor


Evidence

Confidence

Actions

Redraw
