# Supplementary material for: CURTAIN—A unique web-based tool for exploration and sharing of MS-based proteomics data
Source: Proc Natl Acad Sci U S A. 2024 Feb 7;121(7):e2312676121. doi: 10.1073/pnas.2312676121 (PMC10873628; doi:10.1073/pnas.2312676121)
Supplement: Supplementary file 9 — Code S01 (ZIP) [file pnas.2312676121.sd08.zip › Alessi-Lab-curtain-353715d/src/app/components/volcano-plot/volcano-plot.component.html]

Significant Cutoff

Log2 Fold Change Cutoff

Max X axis

Min X axis

Max Y axis

Min Y axis

Plot Title

Marker Size

Custom Popup Label Column

Default
{{col}}
Changes here will only affect newly added annotation ids.

Set Background Data Points Grey

scattergl backend

Volcano Plot Actions

Redraw Plot
Custom FDR Curve
Custom Legend Color
Annotation Text Editor
Clear Selections
