# Supplementary material for: CURTAIN—A unique web-based tool for exploration and sharing of MS-based proteomics data
Source: Proc Natl Acad Sci U S A. 2024 Feb 7;121(7):e2312676121. doi: 10.1073/pnas.2312676121 (PMC10873628; doi:10.1073/pnas.2312676121)
Supplement: Supplementary file 10 — Code S02 (ZIP) [file pnas.2312676121.sd09.zip › Alessi-Lab-curtainPTM-4e27155/src/app/components/protein-information/protein-information.component.html]

**Functional Description:** {{\_data["Function [CC]"].replace("FUNCTION: ", "")}}

| Start | End | Domain name |
| --- | --- | --- |
| {{d.start}} | {{d.end}} | {{d.name}} |

- {{d}}


- {{d}}


- **{{d.position}}** - {{d.note}}


- **{{d.residue}}{{d.position}}** - {{d.disease}}

Data obtained from PTMD
