# Supplementary material for: CURTAIN—A unique web-based tool for exploration and sharing of MS-based proteomics data
Source: Proc Natl Acad Sci U S A. 2024 Feb 7;121(7):e2312676121. doi: 10.1073/pnas.2312676121 (PMC10873628; doi:10.1073/pnas.2312676121)
Supplement: Supplementary file 10 — Code S02 (ZIP) [file pnas.2312676121.sd09.zip › Alessi-Lab-curtainPTM-4e27155/src/app/components/ptm-position-viewer/ptm-position-viewer.component.html]

Modification types for UniProt


{{m}}

Select Databases


{{d.name}}

{{a}}


{{o}}

Redraw Plot

Download as SVG

| Experimental Data Aligned Position (Original Position) | {{g.name}} |
| --- | --- |
|  |  |
| --- | --- |
| {{d.alignedPosition+1}} ({{d.actualPosition+1}}) Kinase Library | {{g.x[d.alignedPosition].split(".")[0]}} ({{alignedPosition[d.alignedPosition][g.name].actualPosition+1}})   {{k.kinase}}   {{d.disease}} |

PhosphoSitePlus link for {{uni["Entry"]}}
