# Supplementary material for: CURTAIN—A unique web-based tool for exploration and sharing of MS-based proteomics data
Source: Proc Natl Acad Sci U S A. 2024 Feb 7;121(7):e2312676121. doi: 10.1073/pnas.2312676121 (PMC10873628; doi:10.1073/pnas.2312676121)
Supplement: Supplementary file 10 — Code S02 (ZIP) [file pnas.2312676121.sd09.zip › Alessi-Lab-curtainPTM-4e27155/src/app/components/qrcode-modal/qrcode-modal.component.html]

QR Code

Scan the QR code to open the link on your mobile device.

Mobile devices with low total system RAM or low amount of allocatable RAM for web process (iOS) might have problem with loading a full dataset in CurtainPTM

Close
