# Supplementary material for: CURTAIN—A unique web-based tool for exploration and sharing of MS-based proteomics data
Source: Proc Natl Acad Sci U S A. 2024 Feb 7;121(7):e2312676121. doi: 10.1073/pnas.2312676121 (PMC10873628; doi:10.1073/pnas.2312676121)
Supplement: Supplementary file 10 — Code S02 (ZIP) [file pnas.2312676121.sd09.zip › Alessi-Lab-curtainPTM-4e27155/src/app/components/raw-data/raw-data.component.html]

| UID | Residue | Position in Protein | Fold Change | Significant | Sequence Window | Peptide Sequence | Localization Score | Actions |
| --- | --- | --- | --- | --- | --- | --- | --- | --- |
|  |  |  |  |  |  |  |  |  |
| --- | --- | --- | --- | --- | --- | --- | --- | --- |
| {{d[dataService.differentialForm.primaryIDs]}} | {{positionMap[d[dataService.differentialForm.primaryIDs]].residue}} | {{d[dataService.differentialForm.position]}} | {{d[dataService.differentialForm.foldChange]}} | {{d[dataService.differentialForm.significant]}} | {{s.slice(0, (s.length-1)/2)}} {{s[(s.length-1)/2]}} {{s.slice((s.length+1)/2, s.length)}} | {{d[dataService.differentialForm.peptideSequence].slice(0, d[dataService.differentialForm.positionPeptide]-1)}} {{d[dataService.differentialForm.peptideSequence][d[dataService.differentialForm.positionPeptide]-1]}} {{d[dataService.differentialForm.peptideSequence].slice(d[dataService.differentialForm.positionPeptide], d[dataService.differentialForm.peptideSequence].length)}} | {{d[dataService.differentialForm.score] \| number}} | A Toggle Bar Charts Volcano plot annotation  Include in profile comparison |
| No raw data available for this protein. |
