# Supplementary material for: CURTAIN—A unique web-based tool for exploration and sharing of MS-based proteomics data
Source: Proc Natl Acad Sci U S A. 2024 Feb 7;121(7):e2312676121. doi: 10.1073/pnas.2312676121 (PMC10873628; doi:10.1073/pnas.2312676121)
Supplement: Supplementary file 10 — Code S02 (ZIP) [file pnas.2312676121.sd09.zip › Alessi-Lab-curtainPTM-4e27155/src/app/components/user-ptm-import-management/user-ptm-import-management.component.html]

##### PTM Data Import

Dataset Name

Upload File (tabulated txt)

The first column must contain uniprot accession id, second column is the numeric position of the PTM in the protein sequence third column is the single amino acid symbol for the residue at that position

Import Database

##### Imported Data

| Name | Actions |
| --- | --- |
| {{d}} | Delete |

Close
